# Towards a quality control framework for cerebral cortical organoids

**DOI:** 10.1038/s41598-025-14425-x

**Published:** 2025-08-11

**Authors:** Héloïse Castiglione, Lucie Madrange, Camille Baquerre, Benoît Guy Christian Maisonneuve, Thomas Lemonnier, Jean-Philippe Deslys, Frank Yates, Thibault Honegger, Jessica Rontard, Pierre-Antoine Vigneron

**Affiliations:** 1 SupBiotech, Ecole d’Ingénieurs en Biotechnologies, Villejuif, France; 2 Commissariat à l’Energie Atomique et aux Energies Alternatives (CEA), Service d’Etude des Prions et des Infections Atypiques (SEPIA), Université Paris-Saclay, Fontenay-aux-Roses, France; 3 NETRI, Lyon, France

**Keywords:** Cerebral organoids, Quality control, Hierarchical scoring methodology, Reproducibility, Standardization., Induced pluripotent stem cells, Cellular neuroscience, Cell fate and cell lineage, High-throughput screening

## Abstract

Cerebral organoids offer significant potential for neuroscience research as complex in vitro models that mimic human brain development. However, challenges related to their quality and reproducibility hinder their reliability. Discrepancies in morphology, size, cellular composition, and cytoarchitectural organization limit their applications, particularly in disease modeling, drug screening, and neurotoxicity testing. Critically, current methods for organoid characterization often lack standardization, restricting their broader applicability. To address the need for standardized quality assessment of cerebral organoids, we developed a Quality Control (QC) methodology for 60-day cortical organoids, evaluating five key criteria using a scoring system: morphology, size and growth profile, cellular composition, cytoarchitectural organization, and cytotoxicity. We implemented a hierarchical approach, beginning with non-invasive assessments to exclude low-quality organoids, while reserving in-depth analyses for those that passed the initial evaluation. To validate this framework, we exposed 60-day cortical organoids to graded doses of hydrogen peroxide (H_2_O_2_), inducing a range of quality outcomes. The QC system demonstrated its robustness by accurately discriminating organoid qualities. Our proposed QC framework is designed to be user-friendly, flexible, and broadly applicable, making it suitable for routine assessment of cerebral organoid quality. Additionally, its scalability enables industrial applications, offering a valuable tool for advancing both fundamental and pre-clinical research.

**Supplementary Information:**

The online version contains supplementary material available at 10.1038/s41598-025-14425-x.

## Introduction

Cerebral organoids have emerged as innovative tools in neuroscience by providing biologically relevant in vitro models that recapitulate aspects of the human brain development and function. These three-dimensional (3D) structures, derived from the neuroectodermal differentiation of pluripotent stem cells, self-organize into complex architectures recapitulating certain regions of the human brain^1^, such as the forebrain, midbrain, hindbrain, or even more specifically the hippocampus, cortex, or choroid plexus^2–12^. Non-specific differentiation protocols can also give rise to unguided whole-brain organoids^13^.

Unlike traditional 2D cultures or simpler 3D models such as spheroids and neurospheres, cerebral organoids recreate a physiologically relevant cellular microenvironment. This complexity enhances cell-cell and cell-matrix interactions, fostering improved differentiation and maturation^14^. While human brain organogenesis remains a highly complex process, tightly regulated both on a spatial and a temporal scale^15^, cerebral organoids have proven their ability to model key neurodevelopmental aspects, including neurogenesis, neuronal migration, neuromorphogenesis, and synaptogenesis^1,15^. Furthermore, transcriptomic and epigenetic analyses have revealed that these models closely mimic developmental trajectories observed in the human fetal brain^15,16^. When derived from patient-specific cells, or when combined with advanced genetic engineering techniques, such features have made cerebral organoids powerful tools for studying neurodevelopmental disorders, such as microcephaly^13^ and trisomy 21^17–19^, as well as for studying neurological cancers^20^, and can also give clues about the pathogenesis of neurodegenerative diseases, including Alzheimer’s disease^21,22^, Parkinson’s disease^23^, and Creutzfeldt-Jakob disease^24^.

Beyond modeling diseases, cerebral organoids have shown promise in neurotoxicity studies^25–27^. Notably, the developing human brain is highly susceptible to environmental insults, and exposure to pollutants or chemicals during pregnancy can disrupt its physiological development. Organoids could provide an unprecedented human-based predictive model to study developmental neurotoxicity (DNT) in response to drugs, chemicals, and pollutants. Studies using cerebral organoids have already explored the effects of valproic acid^28–35^, nicotine^36^, cannabis^37^, bisphenol S^38,39^, cadmium^40^, and nanoplastics^41^, among others^42–45^.

Despite their potential, cerebral organoids face significant challenges regarding quality and reproducibility. Morphological inconsistencies, variations in size, differences in cellular composition or cytoarchitectural organization, and discrepancies in functional activities often arise from the stochastic nature of stem cell differentiation and the spontaneous self-organization occurring within the organoids^1,46^. For instance, within a batch of cerebral organoids, some organoids will display optimal morphology, with dense overall structure and well-defined borders, while others may be poorly compact and will tend to degrade over time by losing cells^47,48^. Moreover, some organoids will exhibit expected cell types and cytoarchitectural organization, whereas others may present disorganized structures and lower proportions of some cell types. Similarly, suboptimal cystic cavities can also be present within some organoids or protrude from their surface^47^. In addition, a necrotic core can also arise in certain organoids^1,46,49^. Non-cerebral structures might also occasionally occur, including germ layers other than neuroectoderm, especially in the unguided organoids^1,50^. These inconsistencies compromise the reproducibility of scientific results, particularly in disease modeling, neurotoxicity testing, and preclinical drug screening, where high-quality and consistent models are essential^46^. Furthermore, this variability is exacerbated by the lack of standardized criteria for organoid generation, culture, and characterization, and the widespread use of in-lab adaptations, leading to varying quality standards between research groups and creating barriers to their broader adoption in industrial and preclinical applications.

Current methods for organoid characterization, including immunohistochemistry^2,13^, transcriptomic profiling^6^, electrophysiological recording^51^, and cytotoxicity studies^52–56^ are valuable but often lack standardization and face several limitations. Many current approaches rely on qualitative and subjective assessments that might introduce inconsistencies and bias. It is common that for daily evaluation of cerebral organoids, researchers rely on morphological observations to assess quality, but this qualitative readout is not frequently detailed in research publications. Although morphological criteria are often used and provide valuable information, their translation into standardized quantitative indicators transferable between laboratories remains partially done, even if recent publications highlight a growing interest in leveraging these criteria as reliable, non-invasive readouts for characterizing cerebral organoids^57–59^. Moreover, some analysis methods commonly used in 2D cell cultures are difficult to transpose to 3D cultures, further complicating the standardization of their characterization^56,60^. Overall, there is a notable lack of robust and well-defined quantitative methodologies for 3D organoid characterization. This gap limits the ability to objectively evaluate cerebral organoids in terms of quality, especially across diverse research groups, ultimately affecting the reliability and consistency of results.

Additionally, this challenge is amplified by the diversity of cerebral organoid types, which depend on the differentiation protocols such as regionalized or unguided whole-brain organoids^1^, and the variety of studies, including disease modeling and neurotoxicological evaluation, each requiring different quality standards and pathophysiological phenotypes. Similarly, the long-term cultures of cerebral organoids, ranging from several months to years, also imply different maturation stages associated with specific characteristics, markers and quality criteria^1,15,61^.

In this study, we propose a Quality Control (QC) framework for 60-day cortical organoids to address these challenges in their evaluation. This system integrates five critical criteria: (A) Morphology, (B) Size and Growth Profile, (C) Cellular Composition, (D) Cytoarchitectural Organization, and (E) Cytotoxicity, into a standardized scoring methodology. The framework is designed hierarchically, prioritizing early, non-invasive evaluations to efficiently exclude organoids of low quality, while reserving in-depth analyses for organoids that have met initial thresholds. To validate its reliability and applicability, we exposed 60-day cortical organoids to gradual doses of hydrogen peroxide (H_2_O_2_), known to cause oxidative stress-induced cell death at non-physiological doses^62,63^, thus producing varying quality levels to rigorously test the scoring system.

The QC methodology we are proposing has been specifically adapted to cortical organoids cultured in vitro for 60 days. For cortical differentiation, 60 days is a critical intermediate stage between so-called immature and mature cortical organoids, characterized by the presence of neural progenitors, as well as neurons and astrocytes^61^. This stage also frequently coincides with the presence of rosette structures that model the development of the neural tube^15,61^, providing key information for studies on neurodevelopmental processes and toxicity in particular.

By minimizing observer bias and enabling objective, reproducible quality assessments, this QC framework enhances the consistency and comparability of results in cerebral organoid research. Moreover, its potential to support both academic studies and industrial scalability highlights its value as a versatile tool for advancing biomedical research.

## Results

### Quality control enables classification of 60-day cortical organoids by quality level

**Fig. 1:**
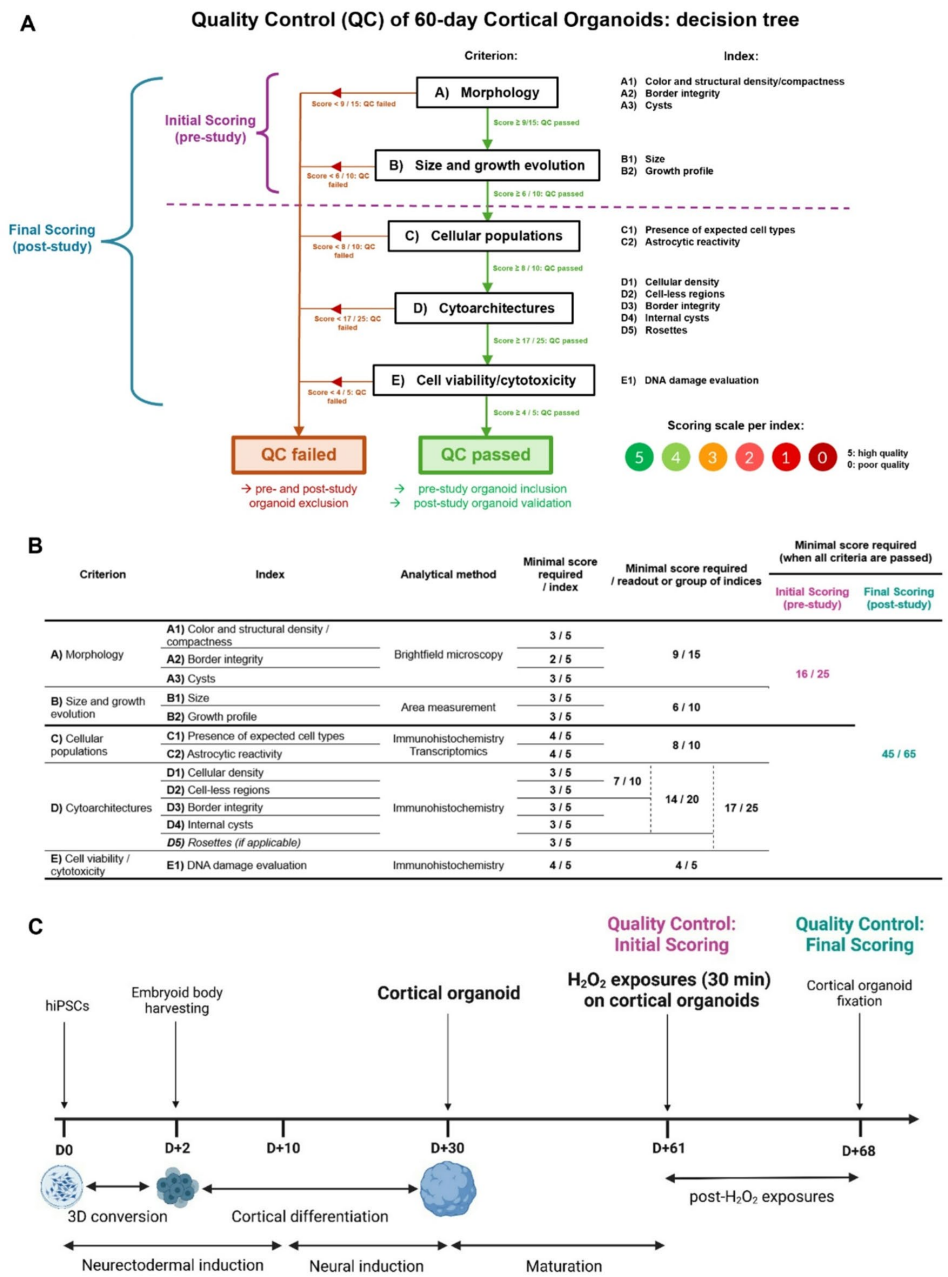
Overview of Quality Control methodology for 60-day cortical organoids, and validation by scoring of H_2_O_2_-exposed cortical organoids. (**A**) Overview of the Quality Control (QC) adapted to 60-day cortical organoids. The QC relies on several criteria, subdivided into indices, for cortical organoid analysis, including Morphology, Size and growth profile, Presence of expected cellular populations at 60 days, Cytoarchitectural organization, and Cellular viability and cytotoxicity levels. For each index, a scoring system enables the evaluation of organoids based on the attribution of scores ranging from 0 (poor quality) to 5 (high quality). The QC follows a hierarchical structure: criteria are assessed sequentially, and failure to meet an initial criterion automatically classifies the organoid as low quality and subsequent criteria are not assessed. The scoring system is divided into two QC: (1) an Initial Scoring to select cortical organoids before entering a study, based on the first two non-invasive criteria Morphology and Growth; and (2) a Final Scoring for complete analysis of cortical organoids based on all the criteria at the end of a study. (**B**) Summary table of QC criteria and minimal scores required per index, and per composite groups of indices and readouts, that have to be obtained for cortical organoids to pass the QC. (**C**) Timeline of cortical organoid generation and culture protocols, including an overview of H_2_O_2_ exposure conditions. Created with BioRender.com (accessed in November 2024). Before H_2_O_2_ exposure at D + 61, cortical organoids were selected based on the Initial Scoring for QC. Exposed cortical organoids at D + 68 were then evaluated following the Final Scoring for complete QC.

We developed a comprehensive QC framework based on a scoring methodology adapted to 60-day cortical organoid evaluation and classification (Fig. 1). This QC scoring system is structured around five primary criteria (A to E) corresponding to cortical organoid analysis readouts – (A) Morphology, (B) Size and growth profile, (C) Cellular composition, (D) Cytoarchitectural organization, and (E) Cytotoxicity level – each further subdivided into specific indices (Fig. 1). For each index, cortical organoids are evaluated on a scale of scores between 0 (low quality) and 5 (high quality). To streamline the process, the criteria are hierarchically organized, prioritizing non-invasive and critical assessments (Fig. 1A). Thresholds with minimum scores are defined for each criterion (Fig. 1B), and failure to meet these thresholds halts further QC evaluation, categorizing the organoid as low-quality and resulting in its exclusion from the study. In cases where all minimal scores are achieved for a criterion, additional composite thresholds, integrating multiple indices, are applied to ensure a robust quality classification (Fig. 1B). For a detailed, illustrated and easy-to-use version of the QC scoring, see Fig. S1 in the Supplementary Information.

This scoring system is designed for two applications: (1) an Initial QC, which relies exclusively on the first two non-invasive criteria (A and B) to determine eligibility of the organoids before entering a study (pre-study QC), and (2) a Final QC based on all the scoring criteria for a complete analysis at the end of a study (post-study QC) (Fig. 1A). Minimal thresholds have also been determined for passing the Initial and Final QC (Fig. 1B).

To validate this QC scoring methodology, we subjected cortical organoids at 60 days of culture to increasing doses of hydrogen peroxide (H₂O₂), a chemical known to cause oxidative stress-induced cellular death^62,63^, to generate organoids with varying quality levels (Fig. 1C). In this context, organoids were first selected for the H₂O₂ treatment experiment within a batch of cortical organoids, using the Initial QC method. H₂O₂ exposures were followed by a recovery period of one week, after which the exposed and non-exposed cortical organoids were evaluated for post-treatment quality using the complete Final QC.

### Initial quality control scoring streamlines the selection of cortical organoids based on non-invasive criteria

**Fig. 2:**
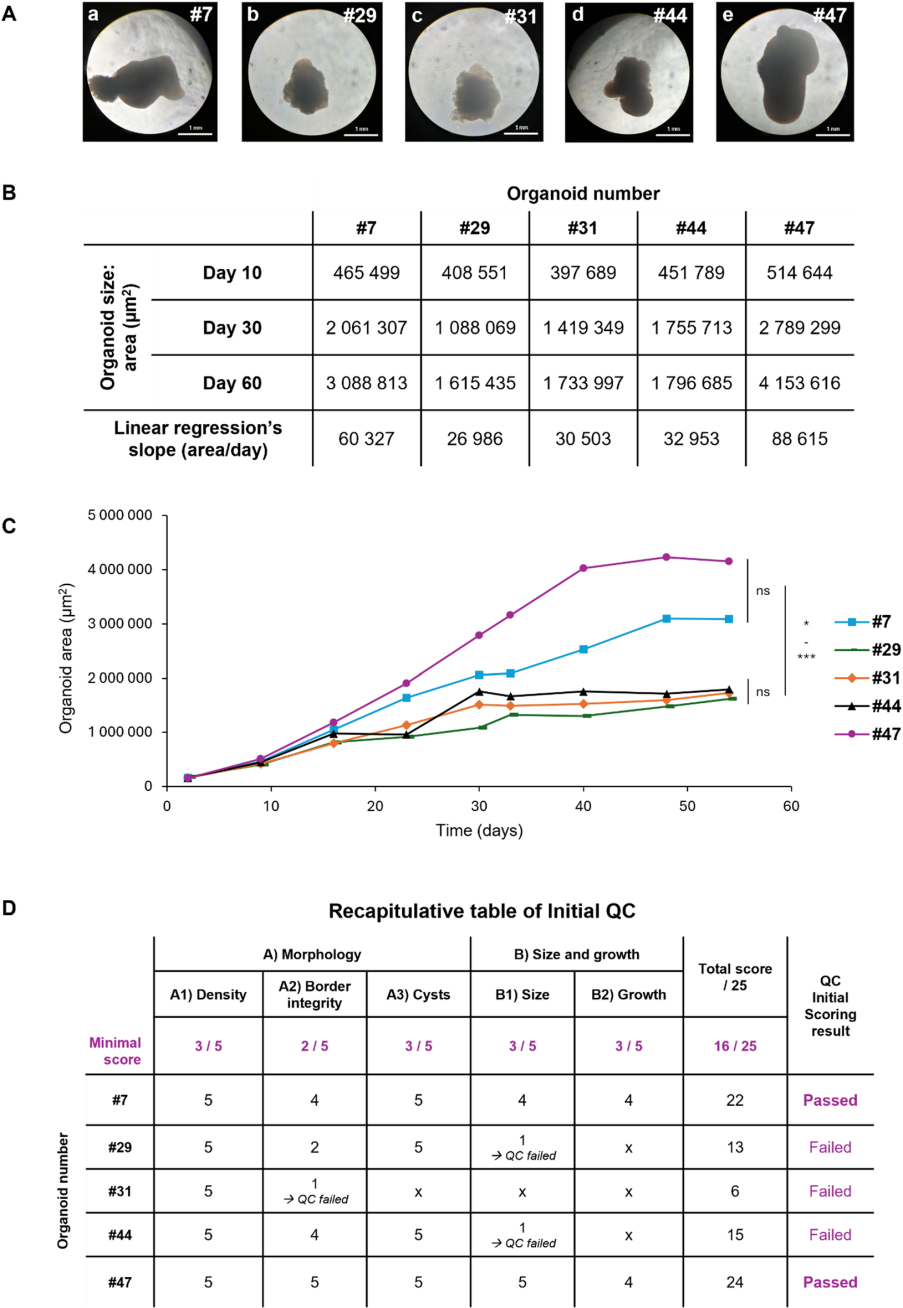
Quality Control (QC) for a selection of cortical organoids before the H_2_O_2_ exposure experiment, following the Initial Scoring based on the first two non-invasive criteria Morphology and Growth profile. (**A**) Morphology of cortical organoids within a batch at 60 days of culture (brightfield, 5X). (**B**) Table summarizing organoid sizes as surface areas at three timepoints of interest (Day 10, Day 30, and Day 60), as well as slopes of corresponding linear regressions. (**C**) Growth curves of individual organoids from D + 2 to D + 60 of culture. Organoids #29, #31, and #44 display an impaired growth profile significantly different from organoids #7 and #47. Friedman test; *n* = 4; p_31 *vs.* 47_ = 0.0175; p_29 *vs.* 27_ = 0.0287; p_44 *vs.* 7_ = 0.0006. (**D**) Recapitulative table of scores obtained by the organoids for each readout and index of the Initial QC. Minimal scores per index and the total minimal score required for Initial QC validation are mentioned. Results of QC for each organoid are indicated as Passed/Failed.

By day 60 of culture, organoids exhibited spontaneous variability in quality due to the intrinsic heterogeneity and stochasticity of differentiation within organoids. Consequently, we evaluated cortical organoids through the Initial QC based on morphology and size evolutions, to select those eligible for the H₂O₂ exposure experiment (Fig. 2). Regarding the morphology evaluation (criterion A), the first index, A1, referring to organoid density and compactness, consistently achieved maximum scores of 5/5 across all the organoids (Fig. 2A, D). On the contrary, discrepancies were observed between the organoids for the second index A2 related to border integrity, with organoid #47 achieving the highest score of 5/5 (Fig. 2Ae, Fig. 2D), organoids #7 and #44 obtaining a score of 4/5 due to the presence of an area with less-defined border (Fig. 2Aa, d, Fig. 2D), and organoid #29 reaching a low score of 2/5 because of poorly-defined borders, but sufficient to pass the QC index (Fig. 2Ab, Fig. 2D). However, organoid #31 failed to reach the minimum required score for border integrity (Fig. 2Ac, Fig. 2D), and was excluded from further analysis. Additionally, no cyst formation was observed, allowing all organoids to pass this third index A3 (Fig. 1A, D).

Regarding the organoid sizes and growth evolutions (criterion B), organoids #29 and #44 were excluded due to insufficient growth both at day 60 and throughout the culture period (Fig. 2B, C, D). Interestingly, organoid #31 would have failed this QC step as well but it had already been excluded based on the first criterion, emphasizing the relevance of this hierarchical order for QC evaluations. Consequently, only organoids #7 and #47 satisfied the minimum thresholds for the two non-invasive criteria and successfully passed the Initial QC (Fig. 2D). Overall, out of 58 cortical organoids generated within this batch, 10 were excluded due to a score lower than 16/25, representing 17% of the total population.

### Final quality control scoring effectively evaluates cortical organoids with varying quality levels

**Fig. 3:**
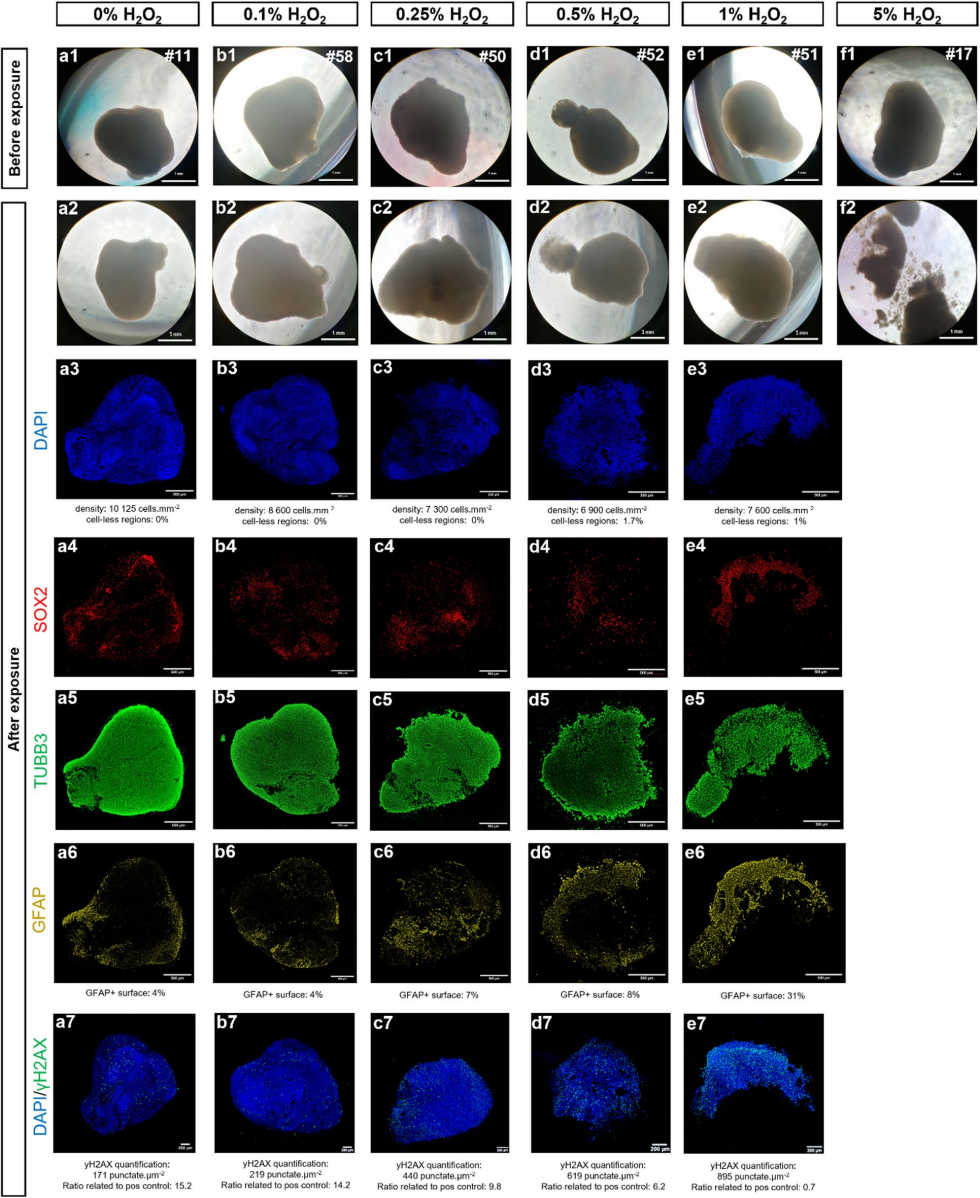
Quality Control (QC) for evaluation of cortical organoids after H_2_O_2_ exposures, following the Final Scoring based on all the criteria. H_2_O_2_ exposures on cortical organoids serve as examples of varying organoid quality levels through incremental H_2_O_2_ doses. (**a1-f1**) Examples of cortical organoids exposed to different H_2_O_2_ doses ranging from 0% to 5%. Morphology before (**a1-f1**) and after (**a2-f2**) H_2_O_2_ exposures serve to evaluate the first criterion related to morphological quality evaluation (brightfield, 5X). Immunofluorescent staining for DAPI (**a3-e3**), neural progenitor marker SOX2 (**a4-e4**), neuronal marker TUBB3 (**a5-e5**), and astrocytic marker GFAP (**a6-e6**) enable the assessment of the following criteria: verification of cell types presence, assessment of astrocytic reactivity, and evaluation of cytoarchitectural organization. Immunofluorescent labeling of DNA damage with ɣH2AX marker enables evaluation of cytotoxicity level (**a7-e7**) (Leica THUNDER microscope, 20X).

**Fig. 4:**
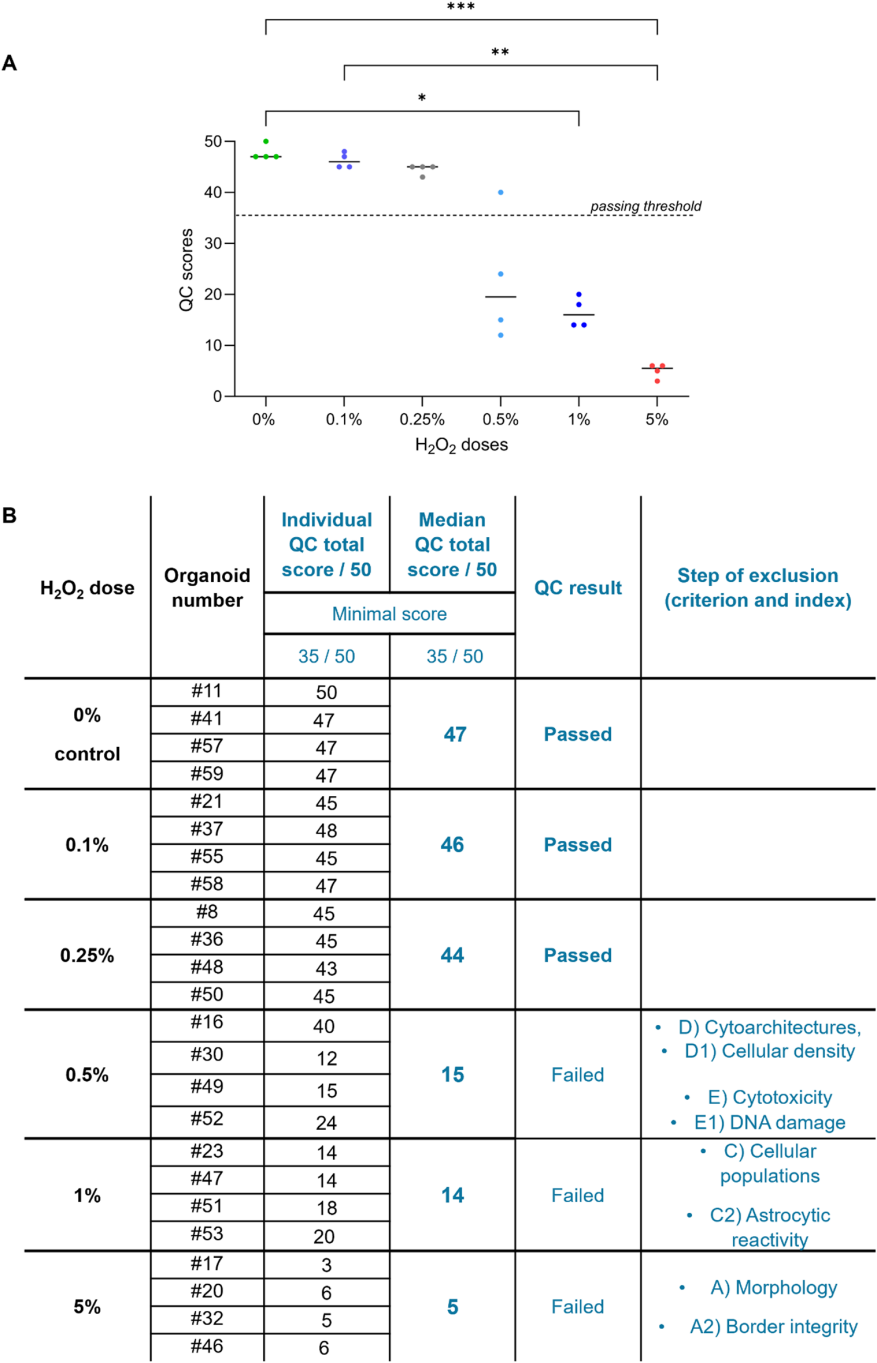
Final QC scores obtained for all the H_2_O_2_-exposed cortical organoids. (**A**) Graphical representation of the final score distributions with median scores for each H_2_O_2_ dose condition, as well as an indication of the QC passing or failure. Scores obtained by control organoids are significantly different from the scores obtained by the 1%- and 5%-treated conditions. Scores obtained by 0.1%-treated condition are also significantly different from the ones obtained by the 5%-treated condition. Kruskal-Wallis test followed by Dunn’s post-hoc; *n* = 4; p_0% *vs.* 1%_ = 0.0353; p_0% *vs.* 5%_ = 0.0007; p_0.1% *vs.* 5%_ = 0.0049. (**B**) Recapitulative table of individual final scores obtained by the H_2_O_2_-exposed organoids, along with minimal scores required for QC validation. Median scores obtained per H_2_O_2_ dose conditions are also mentioned, as well as whether the organoids have passed or failed the QC. For those that have failed the QC, the scoring step at which they have been excluded is specified.

Pre-selected cortical organoids via the Initial QC were included in the H₂O₂ exposure experiment to generate varying degrees of damage (Fig. 3, Fig. S2, Fig. S3). A total of six H₂O₂ concentrations were assayed (*n* = 4 organoids per group): 0% (untreated controls), 0.1%, 0.25%, 0.5%, 1%, and 5% H₂O₂. Before H₂O₂ exposures, all the organoids exhibited an optimal morphology, resulting from the Initial QC selection (Fig. 3a1-f1, Fig. S2a1-i1, Fig. S3a1-i1). After exposures, organoids exposed to 5% of H₂O₂ displayed severe loss of integrity and cellular disaggregation (Fig. 3f2, Fig. S3g2-i2), thus failing to pass the QC at the morphological criterion for the border integrity index (Table 1, Table S1, Fig. 4). This condition also prevented subsequent analyses based on organoid embedding and sectioning for immunostaining, therefore hampering further QC evaluation for these 5% H₂O₂-exposed organoids. Organoids treated with the other H₂O₂ concentrations succeeded in passing the morphology QC according to our criteria (Table 1, Table S1).

**Table 1:** Recapitulative table of H_2_O_2_-exposed organoid for final QC.

H_2_O_2_ dose	Organoid number	A) Morphology			B) Size and growth		C) Cellular populations		D) Cytoarchitectural organization					E) Cellular viability / Cytotoxicity	Total score	QC Final Scoring result
		A1) Density	A2) Border integrity	A3) Cysts	B1) Size	B2) Growth	C1) Expec-ted cell types	C2) Astrocy-tic reactivity	D1) Cellular density	D2) Cell-less regions	D3) Border integrity	D4) Internal cysts	D5) Rosette	E1) DNA damage		
		Minimal score														
		3 / 5	2 / 5	3 / 5	3 / 5	3 / 5	4 / 5	4 / 5	3 / 5	3 / 5	3 / 5	3 / 5	3 / 5	4 / 5	35 / 50	
**0%**	**#11**	5	5	5	NA		5	5	5	5	5	5	NA	5	50	**Passed**
**0.1%**	**#58**	5	5	4			5	5	3	5	5	5		5	47	**Passed**
**0.25%**	**#50**	5	5	5			5	5	3	5	3	5		4	45	**Passed**
**0.5%**	**#52**	5	4	5			5	5	0 *◊ QC failed*	x	x	x		x	24	Failed
**1%**	**#51**	5	3	5			5	0 *◊ QC failed*	x	x	x	x		x	18	Failed
**5%**	**#17**	3	0 *◊ QC failed*	x			x	x	x	x	x	x		x	3	Failed

Recapitulative table of scores for H_2_O_2_-exposed organoid examples assessed through the Final QC. Individual scores obtained for each criterion and index of the QC are indicated, as well as minimal scores required for QC validation. Results of QC for each organoid are indicated as Passed / Failed QC.

The size and growth profile criterion were not reassessed post-H₂O₂ exposures (Table 1, Table S1), as the seven-day recovery period after H₂O₂ treatment was insufficient for meaningful growth analysis.

Subsequent invasive analyses were performed to evaluate cellular composition and cytoarchitectural organization within the organoids (Fig. 3, Fig. S2, Fig. S3). Immunofluorescence staining confirmed the presence of neural progenitors (SOX2), immature neurons (TUBB3), and astrocytes (GFAP) across all remaining conditions (Fig. 3a3-e6, Fig. S2a3-i6, Fig. S3a3-d6), thus validating the QC criterion of cell type presence verification (Table 1, Table S1). However, it must be noted that three organoids (#23, #30 and #47) could not be analyzed by immunolabeling, as they could not be embedded and sectioned with cryostat, likely due to a lack of compactness. Consequently, these organoids were excluded at this QC step (Table S1). Interestingly, they belonged to conditions where all the other organoids failed to pass the QC indices related to cellular composition and cytoarchitectural assessment (Table 1, Table S1).

Regarding the astrocytic reactivity index, GFAP staining in the remaining organoids treated with 1% H₂O₂ (#51 and #53) suggested a high astrocytic reactivity, by covering 31% and 22% of the section area, respectively (Fig. 3e6, Fig. S3d6). This implies potential physiological disruption, thus excluding these organoids according to our QC criteria.

The next index evaluates the overall cellular density, based on DAPI staining analysis. Among the remaining organoids that have passed previous QC steps, it can be observed that the cellular density was notably reduced in organoids exposed with 0.5% H_2_O_2_ (#49 and #52), with densities of 6,900 and 7,600 cells.mm^− 2^, respectively (Fig. 3d3, Fig. S3b3), therefore not reaching the minimal threshold fixed in the scoring (Fig. S1, Fig. 1B) and leading to the exclusion of these organoids.

In contrast, no significant cytoarchitectural disruptions – such as the presence of large cell-less regions, severely altered borders, or occurrence of internal cysts – were observed in the remaining organoids, which therefore met the minimal QC standards for these criteria (Table 1, Table S1). The rosette index was not assessed in this batch, as neurogenic niches were absent in the control organoids (Table 1, Table S1).

Finally, cytotoxicity was evaluated via γH2AX staining, a marker of DNA double-stranded breaks, to quantify DNA damage. For all the remaining organoids exposed to 0%, 0.1% and 0.25% H_2_O_2_, the γH2AX quantification was significantly different from a positive control of maximal γH2AX labeling (Fig. 3a7-c7, Fig. S2a7-i7), therefore passing this final QC step (Table 1, Table S1). Interestingly, all the organoids exposed to higher doses than 0.5% H_2_O_2_, that have been previously excluded at different QC steps, would have failed to pass this final criterion (Fig. 3d7, e7, Fig. S3a7-d7), confirming the validity and relevance of this hierarchical QC system.

Overall, Fig. 4 summarizes the individual final QC scoring results obtained by all the controls and exposed organoids, as well as the median scores reached per condition. We can observe that the unexposed controls and organoids exposed to the doses of H_2_O_2_ at 0.1% and 0.25% successfully passed the QC. However, organoids treated with doses of 0.5%, 1% and 5% H_2_O_2_ failed to pass the QC evaluation. We can notice that these excluded organoids failed at different QC steps, following the hierarchical order of criteria, with 5% H_2_O_2_-exposed organoids excluded during the morphological criterion, 1% H_2_O_2_-exposed organoids failing regarding the cellular composition criterion, and 0.5% H_2_O_2_-exposed organoids rejected through either the cytoarchitectural or the cytotoxicity criteria (Fig. 4B). Similarly, median scores obtained per condition increase incrementally depending on the exposure doses, from a low score of 5/50 obtained for the highest dose of H_2_O_2_, up to an elevated score of 47/50 reached for the unexposed controls (Fig. 4B), thus correlating with the expected damage levels induced by the H_2_O_2_ graded exposures. These results demonstrate the sensitivity of this QC scoring methodology in efficiently and robustly distinguishing organoid quality levels.

## Discussion

The stochastic nature of the differentiation of stem cells and their spontaneous self-organization within cerebral organoids leads to unpredictable variability among them^1^. Consequently, novel approaches have recently emerged to improve culture conditions and enhance organoid reproducibility. Notably, Brain Organoid-on-Chip systems, which rely on the use of microfluidic devices, offer precise fluid flow control and a more physiologically relevant microenvironment^46^, improving cellular viability^36,37,64,65^, neural maturation^64,66^, and organoid homogeneity^64^. In addition, the integration of sensors into microfluidic devices further improves real-time organoid monitoring^27,67^. Flow-based culture systems, including spinning and vertical bioreactors that provide dynamic conditions, enhance differentiation and maturation, by enabling the implementation of mechanochemical, mechanosensing and geometrical transduction effects and better oxygenation in large 3D cultures^68–72^. Additionally, bioengineering strategies like 3D scaffolds and bioprinting further support structural consistency^71,73–76^. Advances also focus on increasing cellular complexity through vascularization^77,78^, incorporation of microglia^79–82^, co-culture systems^83^, and assembloids combining region-specific organoids^84^.

A critical gap remains despite these innovations: the absence of consensus on what defines high-quality cerebral organoids. This lack of standardized metrics or guidelines not only hinders meaningful comparisons across studies but also limits the broader applicability of these models. While complete uniformity should not be the goal, since no living systems are identical, excessive variability in cerebral organoid quality undermines their predictability and reproducibility, potentially leading to inaccurate or unreproducible findings, as well as wasted resources. This challenge is further exacerbated by the absence of standardized protocols for organoid generation, culture and characterization, with numerous in-lab adaptations. This pressing need for the establishment of standardized frameworks in the field has been highlighted in recent publications, which urge reaching a consensus on cerebral organoid nomenclature^85^, and describe an experimental framework for designing, conducting and reporting studies with neural organoids, assembloids and their xeno-transplantations^86^. Altogether, this underscores the necessity for user-friendly and broadly applicable quality control methodologies to ensure cerebral organoid reliability in both academic and industrial applications.

Our scoring-based QC approach, adapted to 60-day cortical organoids, opens the way for a standardized quality control methodology (Fig. S1). By incorporating multiple analysis criteria, including both qualitative macro- and micro-level observations, this framework provides a complete evaluation to lay the foundation for defining what constitutes a high-quality cortical organoid. Importantly, our proposed QC is structured hierarchically to rapidly exclude low-quality organoids, while reserving more detailed analyses for those that passed the initial parameters. This scoring system enables precise evaluation of each index and criterion using tailored examples and scoring scales, covering the full quality spectrum observed in cerebral organoids. While morphological criteria remain qualitative, the clarity and preciseness of the provided examples ensure robust evaluations. These illustrative examples enhance accessibility, allowing both experts and non-specialists to apply the scoring method effectively. For the other criteria, quantitative thresholds have been defined. Minimum scores have been established for each criterion, and failure to meet these scores immediately classifies the organoids as low quality, excluding them from further evaluations (Fig. S1, Fig. 1B). If all the minimum scores are reached, additional thresholds incorporating multiple indices are applied to ensure a thorough quality assessment. As an example, for the morphology criterion, minimal scores required for the three indices are: 3/5 for density, 2/5 for border integrity, and 3/5 for absence of cysts, leading to a total of 8/15. However, the required minimal total score to pass the morphology criterion is not 8/15, but 9/15, implying that the evaluated organoid should not obtain minimal scores for each of the three indices, but should at least reach a higher score for one of them (Fig. 1B).

We demonstrated the effectiveness of our QC method through graded H_2_O_2_ exposures. Due to its ability to induce oxidative stress, eventually leading to cell death through apoptosis and necrosis at high doses^62,63^, H_2_O_2_ has been commonly used in organoid models to study oxidative damage-related mechanisms and to model aging and disease phenotypes^87–90^. For the H_2_O_2_ exposure experiment, organoids were initially selected within a batch of cortical organoids using the Initial QC method. After H_2_O_2_ exposures, untreated and treated organoids were analyzed using the complete Final QC to assess post-treatment quality. Overall, the QC results demonstrated that only the untreated organoids and the organoids exposed to the lower doses of 0.1% and 0.25% H_2_O_2_ passed the QC, while those exposed to higher doses above 0.5% did not (Fig. 4). Median scores reflected the severity of H₂O₂ exposure, ranging from a very low QC score (5/50) for the highest dose to an elevated QC score (47/50) for controls, correlating with the degree of damage caused by the graded H_2_O_2_ exposures. Importantly, failures occurred at different steps of the hierarchical QC process: organoids exposed to 5%, 1% and 0.5% H_2_O_2_ were excluded during the first criterion (morphology), the third criterion (cellular composition), or the fourth criterion (cytoarchitecture), respectively. These results emphasize the necessity of a stepwise evaluation, as certain defects are detectable only through deeper cellular or subcellular analysis. Interestingly, organoids excluded early in the QC process were later found to exhibit high cytotoxicity levels in the final criterion, reinforcing the relevance of this hierarchical QC system. Taken together, these observations demonstrate the precision and reliability of the QC scoring system in differentiating organoid quality levels.

Although extensive characterization and validation are recommended for the development of novel differentiation protocols^86^, including single-cell profiling with mapping to brain atlases or functional characterizations through electrophysiological activity recording, such analyses might be difficult to implement for routine QC. Our proposed QC framework is intentionally designed to be flexible and prioritizes widely adopted and cost-effective techniques, making it suitable for regular assessment of cerebral organoid quality across laboratories without the need for advanced or cost-prohibitive resources.

Interestingly, a few recent publications have demonstrated a growing interest in the use of morphological criteria as reliable non-invasive readouts for the characterization of cerebral organoids^57–59^. Charles and colleagues have implemented a non-invasive quality control system relying on morphological criteria, enabling the classification of evaluated organoids in high- or low-quality categories for organoid pre-selection^59^. Remarkably, they integrated brightfield image processing with machine learning tools, opening the way for automated quality assessment. However, this system is based solely on morphological observations and does not account for other important analysis criteria that may provide valuable insights beyond what is visible at the macro-scale. As our study demonstrates, using a scoring scale enables the detection of subtle variations that a binary classification might overlook. Additionally, the inclusion of parameters such as sphericity can be questioned, as many factors could be at the origin of shape variations independent of organoid quality. Serafini et al. have also developed a non-invasive imaging analysis method for cerebral organoid characterization, also based on brightfield images, and considering several morphological criteria^57^. Using a 3D quantitative phase imaging technique, they assess parameters in a non-invasive manner, such as cellular content, cell morphologies, and rosettes. Similarly, Ikeda and colleagues have implemented a non-invasive morphological characterization of cerebral organoids combined with transcriptomic analyses^58^. Interestingly, some analysis criteria are similar to those we selected, such as verification of transparency level and analysis of cystic structures^58^. More broadly, an increasing number of studies rely on organoid analysis using brightfield imaging coupled with machine learning techniques, underscoring their growing significance in the field^91–94^. Overall, morphological analysis serves as a valuable initial approach for assessing the quality and harvestability^95^ of cerebral organoids. Organoids with low QC scores often exhibit poor compactness, irregular borders or large cysts, making them unsuitable for harvesting and analysis. Our initial QC based on morphology reflects technical harvestability, while final quality is confirmed through more detailed, often invasive, analyses via our final QC.

While our proposed scoring system lays a foundation for organoid QC, there are still opportunities for further refinement and invasive techniques replacement. Notably, this scoring system can be applied manually, as proposed in this study, but could also be automated using image processing and machine learning analysis tools, offering flexibility, increased objectivity, faster execution, and higher throughput. Automated analysis could enable organoid images to be processed and partially scored through computational workflows, reducing variability in evaluations that might arise from individual interpretation. In particular, the automation could be envisaged primarily for criteria A) Morphology, based on brightfield images, as demonstrated by Charles and colleagues^59^, and D) Cytoarchitecture, based on immunofluorescence images.

Additionally, incorporating other non-invasive criteria could significantly enhance the transferability of the scoring system for preclinical applications. These could include the detection of specific markers in the conditioned medium, such as lactate dehydrogenase activity measurement for cytotoxicity evaluation^56^, apoptosis quantification, measurement of reactive oxygen species for oxidative stress analysis, and evaluation of metabolic activity. While numerous ready-to-use kits are available on the market for these analyses, particular attention must be paid to the normalization step, as these kits are typically designed to be normalized by cell number, which cannot be easily determined in 3D cultures^56^. Other non-invasive methodologies also comprise label-free imaging techniques adapted for cerebral organoid characterization in 3D^57,96^.

Although we confirm the presence of expected cell types in the evaluated organoids with the third criterion, it could also be valuable to ensure the absence of non-neural cells originating from non-neuroectodermal lineages during the differentiation^1,50^, particularly through RNA sequencing, provided the feasibility of integrating such transcriptomic analyses into the routine QC workflow.

Regarding the last criterion, which addresses cellular viability and cytotoxicity assessments, we evaluated DNA damage through γH2AX immunolabelling. However, other methods could also replace or complete this example, such as apoptosis detection via cleaved-caspase3 immunolabelling^54^, TUNEL assay^53^, or transcriptomic analyses evaluating pro- and anti-apoptotic markers like BAX and BCL2^52^.

After completing the morphological, structural, and cellular viability characterizations of our proposed QC approach, functional evaluations of the cerebral organoids could also be considered^97,98^. This could involve assessing electrophysiological activity using calcium imaging^13^, patch-clamp^2^, or microelectrode arrays (MEA) recordings^51,99^, to confirm functional quality. Nonetheless, the use of these techniques with 3D organoids still poses some technical challenges, and their relevance depends on the specific applications^97,98^.

Likewise, Organoid Intelligence (OI) is an emerging interdisciplinary field that integrates electrophysiological recordings from cerebral organoids with artificial intelligence-based analysis^100,101^. Although still in its early stages and dependent on further refinement of organoid models^102^, studies have shown that cerebral organoids can exhibit structured electrical activity, suggesting a potential for functional information processing. In this context, OI could enrich QC pipelines by offering insights into the functional maturity of neuronal networks. However, challenges remain, including technical limitations related to 3D cultures and variability in the onset and robustness of organoid activity. Overall, establishing reproducible functional readouts, such as electrical activity thresholds or machine-learning-based classification of network dynamics could considerably enhance scoring systems and may eventually support a new criterion of functional indices to complement existing QC features.

Ultimately, our framework provides flexibility, enabling the inclusion or exclusion of parameters based on the specific characteristics of the study (e.g., neurogenic niches, which could not be assessed here). However, to ensure consistency, it is crucial to define and validate thresholds through preliminary testing, especially when working with specific cell lines. Similarly, growth dynamics should be adjusted according to the number of cells used during 3D seeding.

Although our proposed QC methodology is specifically designed for the assessment of cortical organoids at 60 days, a key stage in their maturation process, our approach remains flexible and adaptable to different types of cerebral organoids and maturation timepoints. It may also serve as a QC framework for studies on disease modeling, where the scoring system could be adapted to focus on specific phenotypes critical for recapitulating pathological hallmarks, as well as in the context of neurodevelopmental toxicity evaluations, where it could facilitate systematic comparisons between exposed and non-exposed organoids. Additionally, reproducibility and scalability of cerebral organoids are central parameters to be considered both in fundamental and preclinical research. Beyond quality evaluation, this proposed scoring methodology can also indirectly reflect the intra- and inter-batch variability by analyzing the score dispersions among organoids within the same batch, and across different batches. In this regard, the QC scores can be used retrospectively to evaluate batch consistency. By addressing these evolving needs, this framework paves the way for more robust, reproducible, and versatile organoid-based research. Notably, it represents a critical step toward the much-needed collaborative effort to define and standardize quality expectations for different types of organoids. As the field moves toward increasingly complex models, such as assembloids^84^, maintaining scientific rigor requires a shared foundation.

## Methods

### HiPSC culture and maintenance

Human induced Pluripotent Stem Cells (hiPSCs) were generated by reprogramming BJ primary foreskin fibroblasts obtained from ATCC (CRL-2522), using non-integrative Sendai virus vectors following the manufacturer’s instructions (A16517, ThermoFisher Scientific). Pluripotency was confirmed by identifying specific pluripotency markers through Reverse Transcriptase-Polymerase Chain Reaction (RT-PCR), and regular tests were conducted to verify the absence of mycoplasma. The culture and maintenance of hiPSCs were performed as previously reported^21,22,56^. Briefly, hiPSCs were maintained on Geltrex-coated cell culture plates (A1569601, Gibco) and cultured in mTeSR Plus medium (100–0276, STEMCELL Technologies) supplemented with 1% Penicillin/Streptomycin (P/S) (15140122, Gibco), at 37 °C in a 5%-enriched CO_2_ atmosphere. hiPSCs were passaged upon reaching 50–70% confluency using 0.02% ethylenediaminetetraacetic acid (EDTA) treatment (E8008, Sigma-Aldrich).

### Generation and culture of cerebral cortical organoids

Cerebral cortical organoids were generated as previously reported^56^, from a protocol adapted from methods described by Xiang et al.^4,5^ relying on dorsal forebrain-regionalized differentiation. On day 0, hiPSCs were detached using 0.02% EDTA treatment and dissociated with Accutase (AT-104, STEMCELL Technologies) to obtain a single-cell suspension. These cells were seeded in V-bottom cell-repellent 96-well plates (651970, Greiner Bio-One) at a density of 20,000 cells/well in neural induction medium containing Dulbecco’s Modified Eagle Medium/Nutrient Mixture F-12, GlutaMAX supplement (DMEM/F-12, 10565018, Gibco), 15% (v/v) KnockOut Serum Replacement (KOSR, 10828010, Gibco), 1% Minimum Essential Medium-Non-Essential Amino Acids (MEM-NEAA, 1140035, Gibco), 1% P/S, 100 nM LDN-193,189 (72147, STEMCELL Technologies), 10 µM SB-431,542 (72232, STEMCELL Technologies), 2 µM XAV-939 (X3004, Sigma-Aldrich), 100 µM β-mercaptoethanol (21985023, Gibco), and supplemented with 5% Fetal Bovine Serum (FBS, 10270106, Gibco) and 50 µM Y-27632 (72304, STEMCELL Technologies). On day 2, embryoid bodies (EBs) were collected and transferred into 24-well suspension cell culture plates (144530, Nunc). The neural induction medium was renewed every two days until day 10, with FBS removed from day 2, and Y-27632 removed from day 4. From day 10 to day 18, EBs were cultured in differentiation medium without vitamin A, containing DMEM/F-12:NeuroBasal Medium (21103049, Gibco) at 1:1 ratio, supplemented with 0.5% (v/v) MEM-NEAA, 1% P/S, 0.5% N2 supplement 100 × (17502-048, Gibco), 1% B-27 supplement minus vitamin A (12587010, Gibco), 1% HEPES solution (H0887, Sigma-Aldrich), 0.025% human insulin (19278-5 mL, Sigma-Aldrich), and 50 µM β-mercaptoethanol. From day 18, EBs were cultured in a differentiation medium with vitamin A, following the same composition as the previously described medium, but replacing the B-27 supplement minus vitamin A, with B-27 supplement with vitamin A (17504044, Gibco), and supplemented with 20 ng/mL BDNF (78005, STEMCELL Technologies), 200 µM ascorbic acid (A9290225G, Sigma-Aldrich), and 200 µM cAMP (73886, STEMCELL Technologies). Cortical organoids were cultured in 24-well plates with 500 µL of culture medium renewed every two days from day 2 to day 28. After day 28, the medium volume was increased to 1 mL and renewed once a week. Organoids were cultured under agitation (80 rpm) at 37 °C, in a 5%-enriched CO_2_ atmosphere.

### H_2_O_2_ exposures on cortical organoids

Cortical organoids were exposed on day 61 of culture to hydrogen peroxide (H_2_O_2_) (1.07209.0250, Supelco) diluted in differentiation medium with vitamin A, at several doses (0.1%, 0.25%, 0.5%, 1% and 5%) during 30 min at 37 °C. After exposure, cortical organoids were washed once with fresh culture medium to remove excess H_2_O_2_, were maintained in culture for 7 days, and were fixed on day 68.

### Quality control of cortical organoids: scoring system

A multi-criteria scoring system was developed for the QC of 60-day cortical organoids (Fig. 1 and Fig. S1). Briefly, each QC criterion is subdivided into indices, which are scored on a scale ranging from 0 (low quality) to 5 (high quality) to assess cortical organoid quality (Fig. S1). To facilitate the evaluation process, criteria are hierarchically organized, with priority given to non-invasive and critical parameters (Fig. 1A). Minimum score thresholds are defined for each individual index (Fig. 1B), and failure to meet any of these minimal scores results in immediate classification as low quality, and exclusion from further analysis (Fig. 1A). Through this design, the QC framework prioritizes and provides more weight to early-stage criteria. Minimum scores were defined not only at the level of individual indices, but also across each criterion. Therefore, even if an organoid meets the minimum scores for all indices within a given criterion, it may still be excluded if the total criterion score falls below the required threshold (Fig. 1A, B).

For the QC scoring, a detailed description (Fig. S1) outlines expected values and scoring thresholds for each index. Additionally, a summary table with the minimum scores required to pass the QC for each criterion is presented (Fig. 1B). Based on their individual QC scores obtained, cortical organoids are classified into “QC passed’ or “QC failed” categories, with the specification of the failed scoring step for any organoid that did not pass the QC. More precisely, the scoring approach is tailored to be usable both for pre- and post-study, referred to as Initial QC and Final QC, respectively.

### Initial quality control

In the pre-study phase, the first two non-invasive criteria, A (Morphology) and B (Size and Growth Profile), are evaluated across a batch of cortical organoids, to identify those suitable for inclusion in the subsequent study. The morphology of the organoids is assessed based on their color, density, compactness, border integrity, as well as depending on the absence or presence of cysts. In addition, organoid sizes and growth profiles are monitored to ensure they remain within expected growth ranges.

### Final quality control

In the post-study phase, all five criteria – A to E – are used to thoroughly validate organoid quality. This includes additional evaluations of cellular populations (criterion C), where the presence of the three expected cell types (neurons, astrocytes, neural progenitors) and astrocytic reactivity are analyzed, as well as assessments of the cytoarchitectural organization (criterion D), including cell density, proportion of cell-less regions, border integrity, presence of neurogenic areas, and occurrence of internal cysts. Finally, cell viability and cytotoxic markers (criterion E) are evaluated, with a focus on DNA damage, to ensure organoids have maintained low cytotoxicity levels throughout the study.

### Longitudinal monitoring of cortical organoid morphology and growth evolution

For cortical organoid morphology and growth profile monitoring over time, brightfield images of the organoids were acquired at regular timepoints during the culture (D + 2, D + 9, D + 16, D + 23, D + 30, D + 33, D + 40, D + 48, D + 54 and D + 61), using a DM IL LED Inverted Laboratory Microscope (Leica Microsystems) (5X). To assess the organoid size, the surface area of the organoid was measured from the brightfield images on FIJI/ImageJ software, version 1.54f^103^.

### Immunohistochemistry

Cortical organoids were fixed in 4% paraformaldehyde (11699408, Q Path) for 2 h at room temperature (RT) under smooth agitation, followed by three washes of 10 min with Phosphate Buffered Saline solution (PBS) (18912-014, Gibco) at RT under smooth agitation, and immersed in 30% (v/v) sucrose (S9378, Sigma-Aldrich) dissolved in PBS for 48 h at 4 °C. The organoids were then transferred in a solution composed of 7.5% (v/v) gelatin (G9391, Sigma-Aldrich) and 15% (v/v) sucrose dissolved in PBS for 1 h at 37 °C, before being embedded in this solution for 15 min at 4 °C. Embedded organoids were then snap-frozen in isopentane (M32631, Sigma-Aldrich) and stored at -80 °C until use. Frozen organoids were sectioned in slices of 20 μm thickness using a cryostat (CM1850 UV, Leica Biosystems). For the immunofluorescent staining, organoid slices were permeabilized and blocked with a solution containing 0.2% Triton X-100 (T-9284, Sigma-Aldrich), 3% bovine serum albumin (BSA, A2153, Sigma-Aldrich), and 1% normal goat serum (NGS, G9023, Sigma-Aldrich) in PBS for 1 h at RT. Then, the slices were incubated with primary antibodies diluted in the blocking solution at 4 °C overnight in a humidified chamber and were washed with 0.2% Triton in PBS three times. Then, organoid slices were incubated with secondary antibodies and 4′,6-diamidino-2-phenylindole (DAPI, dilution 1:1000) for 1 h at RT in a dark humidified chamber and were washed three times with 0.2% Triton in PBS. The slices were mounted using ProLong Gold Antifade Mountant (11539306, Invitrogen), and observed under a Leica THUNDER microscope (THUNDER Imager 3D Assay, Leica Microsystems) with the Leica Application Suite X software version 3.8.2.27713. Primary and secondary antibodies used are listed in Table 2.

**Table 2:** Primary and secondary antibodies used for immunofluorescent stainings.

Primary antibody	Host and isotype	Supplier and reference	Dilution
Anti-SOX2	Mouse IgG1	Proteintech, 66477-1-Ig	1:500
Anti-TUBB3	Mouse IgG2a	Biolegend, 801,202	1:500
Anti-GFAP	Rabbit IgG	Dako, Z0334	1:1000
Anti-γH2AX	Mouse IgG1	Sigma-Aldrich, 05-636	1:500

Secondary antibody	Host	Supplier and reference	Dilution
Alexa Fluor^®^ Cy3 anti-mouse IgG1	Goat	Jackson ImmunoResearch, 115-165-205	1:500
Alexa Fluor^®^ 488 anti-mouse IgG2a	Goat	Jackson ImmunoResearch, 115-547-186	1:500
Alexa Fluor^®^ 647 anti-rabbit IgG	Goat	Jackson ImmunoResearch, 111-605-144	1:500
Alexa Fluor^®^ 488 anti-mouse IgG1	Goat	Jackson ImmunoResearch, 115-547-185	1:500

### Image-based quantifications of cellular density and cell-less regions

Cellular density was calculated based on DAPI positive surface, without considering cell-less zones (“holes”) since this second parameter was considered in the subsequent index. Both quantifications of cellular density and cell-less regions relied on determination of DAPI positive surface and were normalized to the total surface area of the organoid slice. Briefly, the DAPI positive surface was determined using the “Adjust Threshold” function of FIJI/ImageJ. For the cellular density, the threshold was adjusted to correspond with the DAPI labelling, while for the cell-less areas, the threshold was increased to cover the entire surface except the cell-less regions/holes. Estimation of cell number was calculated based on DAPI positive surface, considering an average nucleus area of 80 µm^2^.

### Image-based quantification of GFAP positive surface expression

Glial Fibrillary Acidic Protein (GFAP) positive surface expression was calculated using the “Adjust Threshold” function on FIJI/ImageJ, as previously described in this section, and normalized to the total DAPI positive surface area of the cryosection.

### Image-based quantification of ɣH2AX marker and comparison with a positive control

Quantification was conducted by counting ɣH2AX punctate and by normalizing to the DAPI positive surface expression, across several sections of individual organoids (*n* = 2–3 slices per organoid, *n* = 4 organoids per condition). Images were first binarized on FIJI/ImageJ, then the “Analyze Particles” function was applied with the following parameters: Size (micron2): 15-infinity, Circularity: 0.00–1.00. To statistically compare the maximal ɣH2AX immunolabelling in cerebral organoids with positive controls, the standard deviation of the positive controls was first calculated. Then, the difference between the mean value of the positive controls and the value for the organoids was determined. Finally, this difference was expressed as a ratio relative to the standard deviation of the positive controls.

### Experimental design, randomization and statistical analysis

The QC framework – including criteria, scores, thresholds, and representative organoid examples illustrating each score (Fig. S1) – was developed based on data and images collected from a large number of in-house studies using cortical organoids derived from healthy donor-iPSCs. These studies, conducted over the past four years, encompassed more than 30 independent organoid batches and a total of more than 1200 individual organoids that were characterized. This extensive dataset provided sufficient perspective to discriminate between high- and low-quality organoids, and intermediate stages, as well as to determine which criteria were the most critical in the QC system structure.

Cortical organoids used for the H_2_O_2_ exposure experiment were selected based on their Initial QC scores (harvestability) and were randomly distributed across experimental groups before H_2_O_2_ exposures (*n* = 4 per group). Images and data from the exposed organoids were collected and coded by one experimenter, then blindly evaluated for QC by a second experimenter.

Statistical analyses were conducted using GraphPad Prism 10 (version 10.5.0). Since residuals were not normally distributed (Shapiro-Wilk test), non-parametric tests were used (Friedman and Kruskal-Wallis), followed by Dunn’s post hoc correction for multiple comparisons.

## Supplementary Information

Below is the link to the electronic supplementary material.

Supplementary Material 1

## Data Availability

The datasets generated during and/or analysed during the current study are available from the corresponding author on reasonable request.
