# Supplementary material for: Towards a quality control framework for cerebral cortical organoids
Source: Sci Rep. 2025 Aug 11;15:29431. doi: 10.1038/s41598-025-14425-x (PMC12340003; doi:10.1038/s41598-025-14425-x)
Supplement: Supplementary file 1 — Supplementary Material 1 [file 41598_2025_14425_MOESM1_ESM.docx]

**Supplementary Information**

**Towards a Quality Control Framework for Cerebral Cortical Organoids**

Héloïse Castiglione^1,2,3,*^, Lucie Madrange^1,2^, Camille Baquerre^3^, Benoît Guy Christian Maisonneuve^3^, Thomas Lemonnier^1,2^, Jean-Philippe Deslys^2^, Frank Yates^1,2^, Thibault Honegger^3^, Jessica Rontard^3,&^ and Pierre-Antoine Vigneron^1,2,&,*^

1: SupBiotech, Ecole d’Ingénieurs en Biotechnologies, Villejuif, France.

2: Université Paris-Saclay, Commissariat à l’Energie Atomique et aux Energies Alternatives (CEA), Service d’Etude des Prions et des Infections Atypiques (SEPIA), Fontenay-aux-Roses, France.

3: NETRI, Lyon, France.

&: These authors contributed equally

*: Correspondence: heloise.castiglione@netri.com ; pierre-antoine.vigneron@supbiotech.fr

**
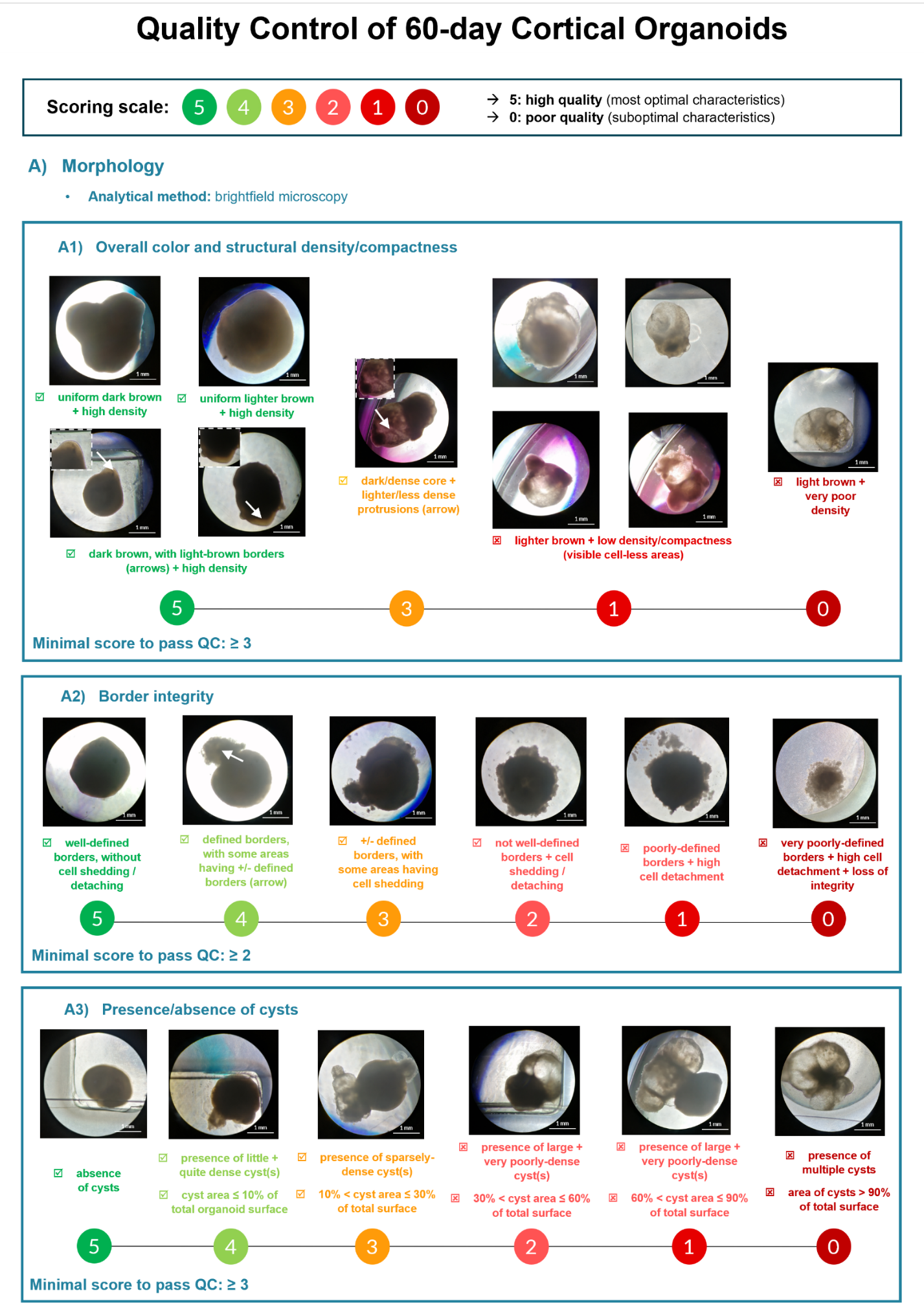
**

**
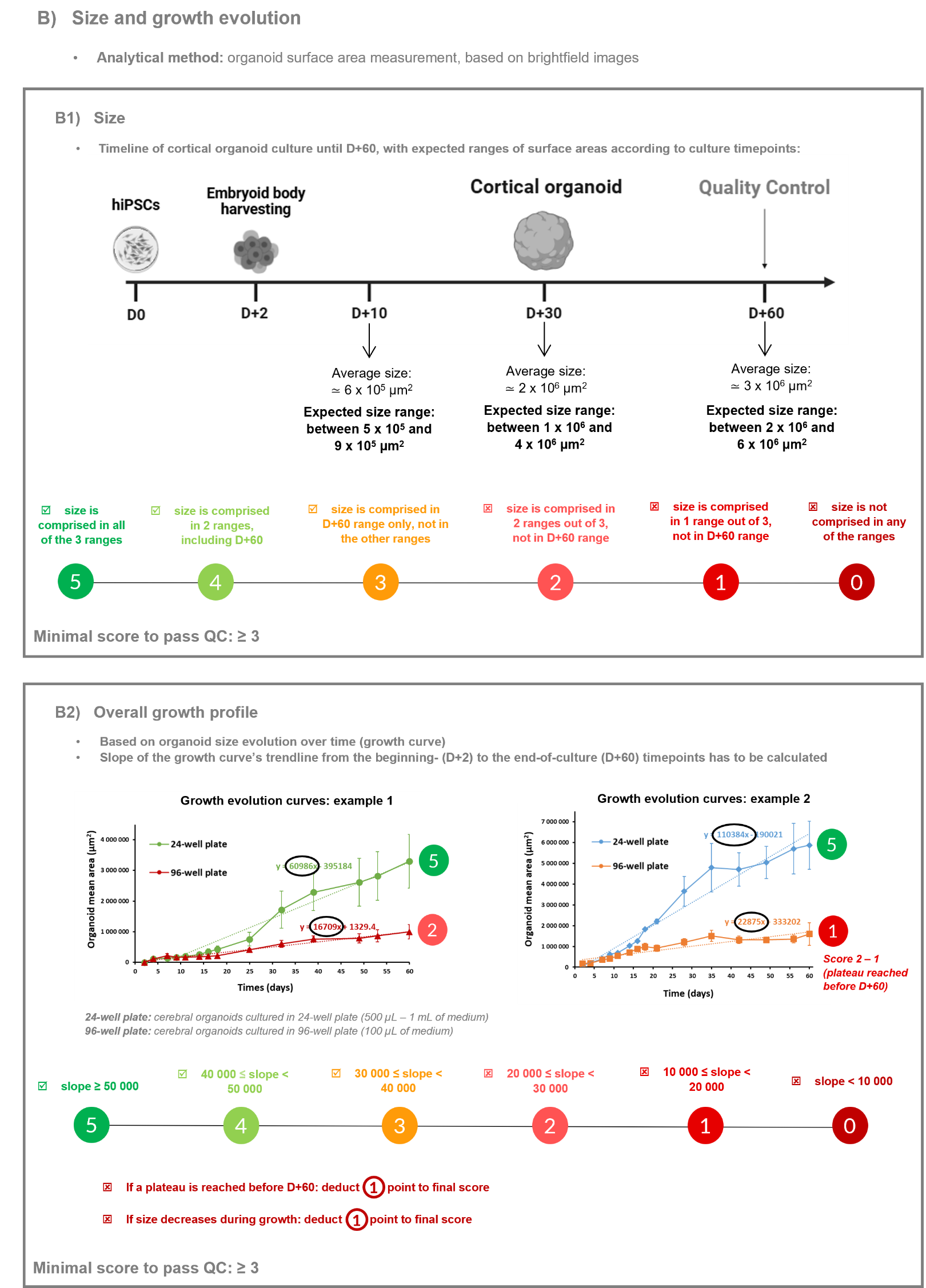
**

**
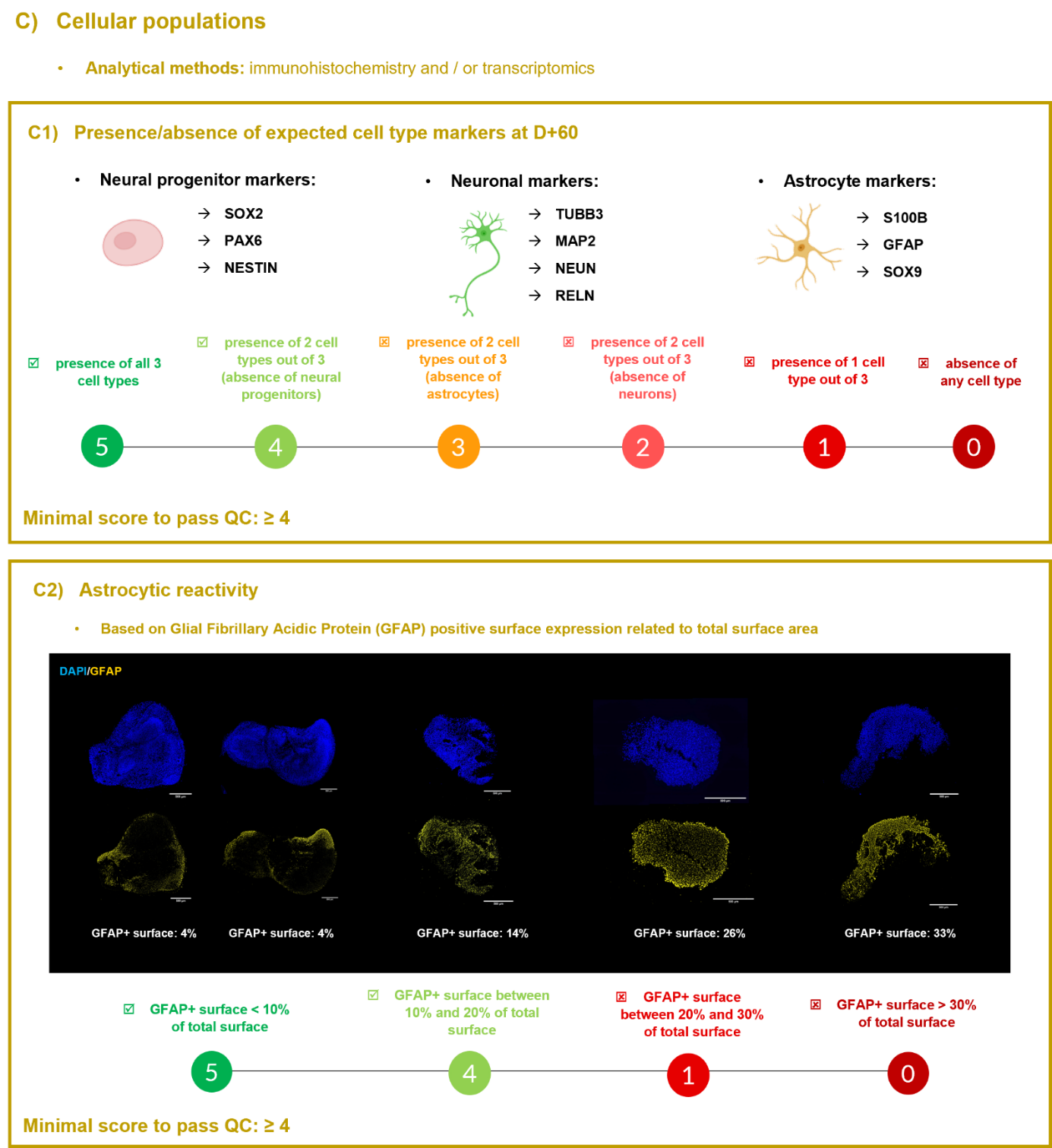
**

**
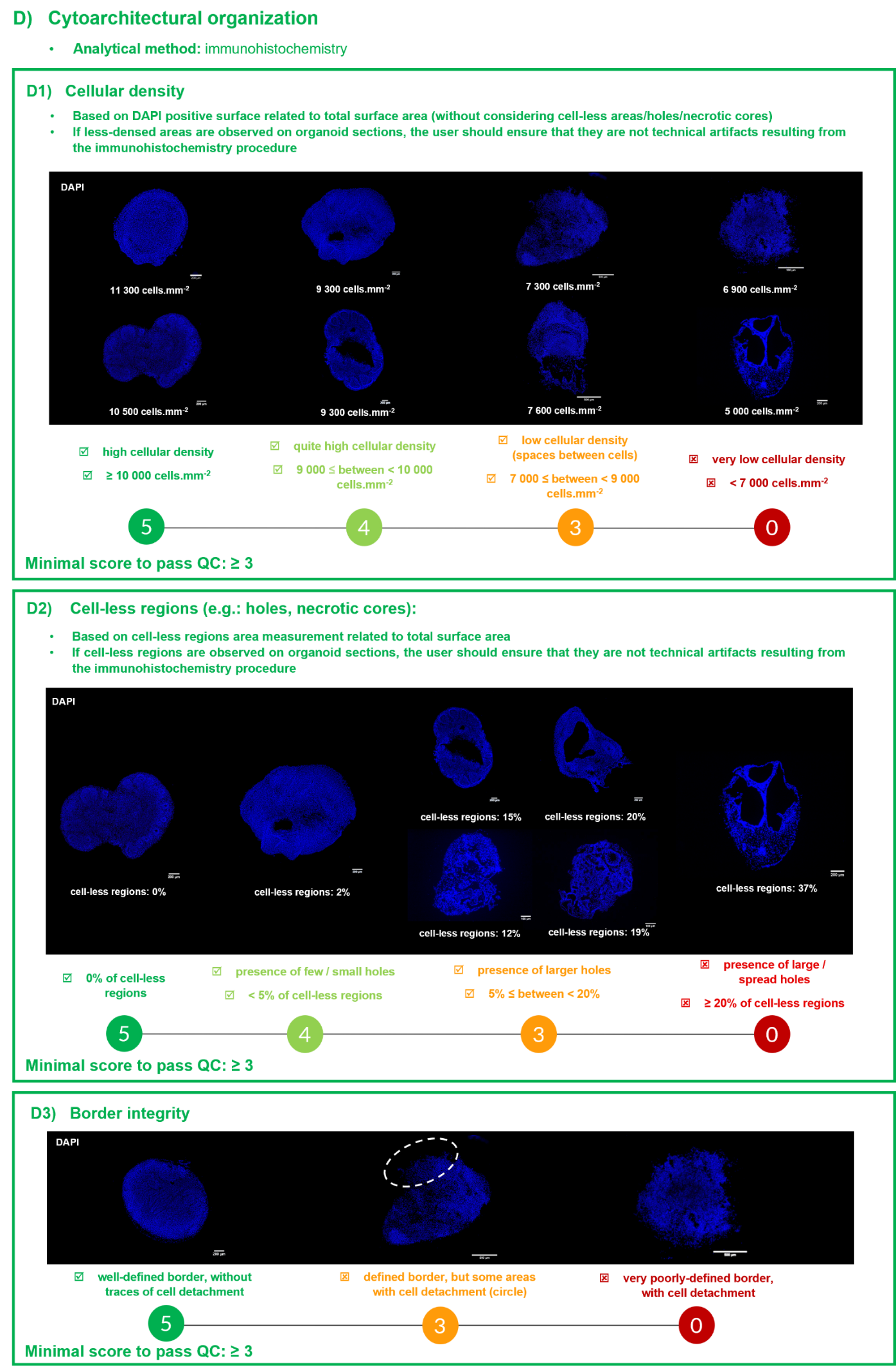
**

**
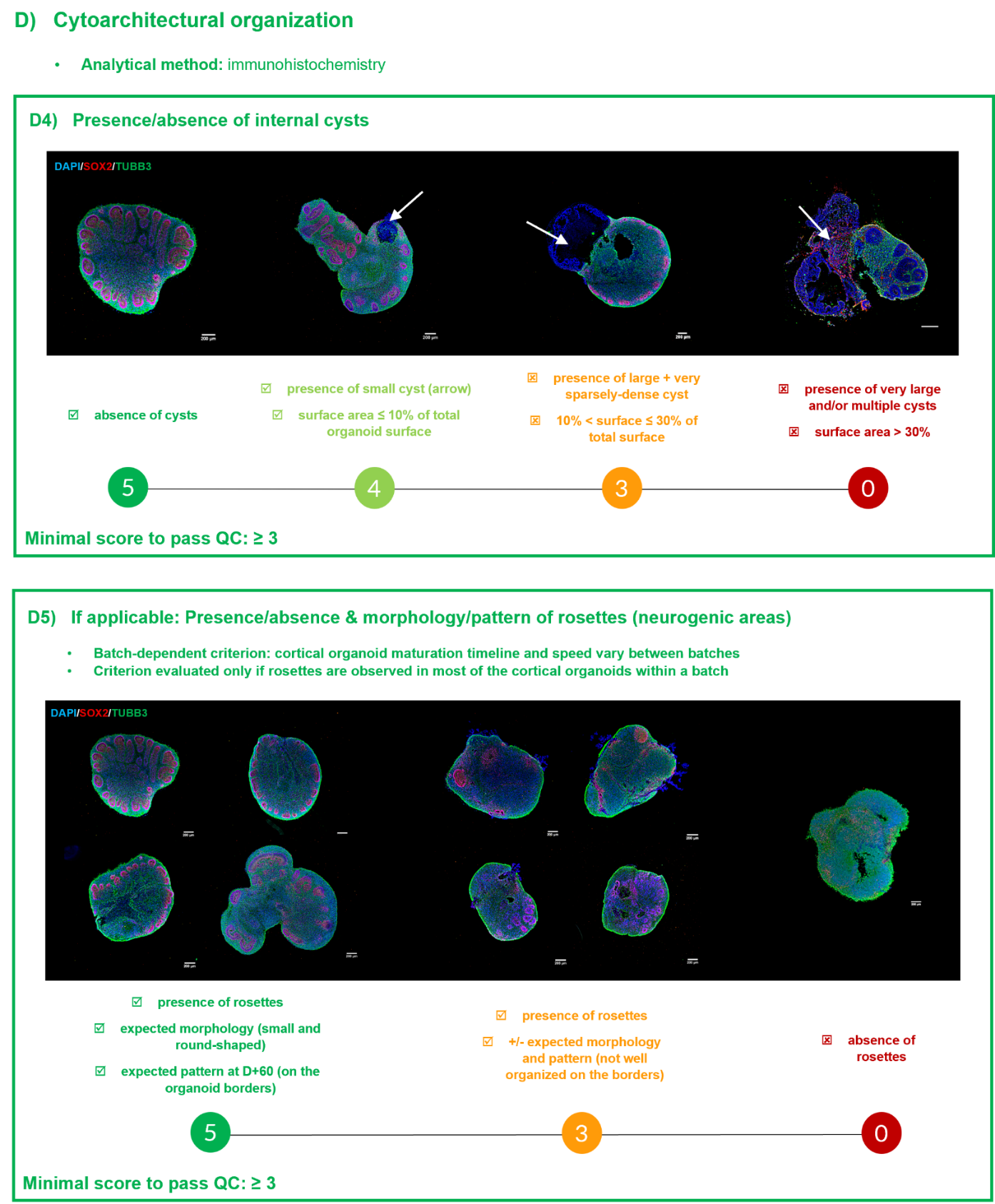
**

**
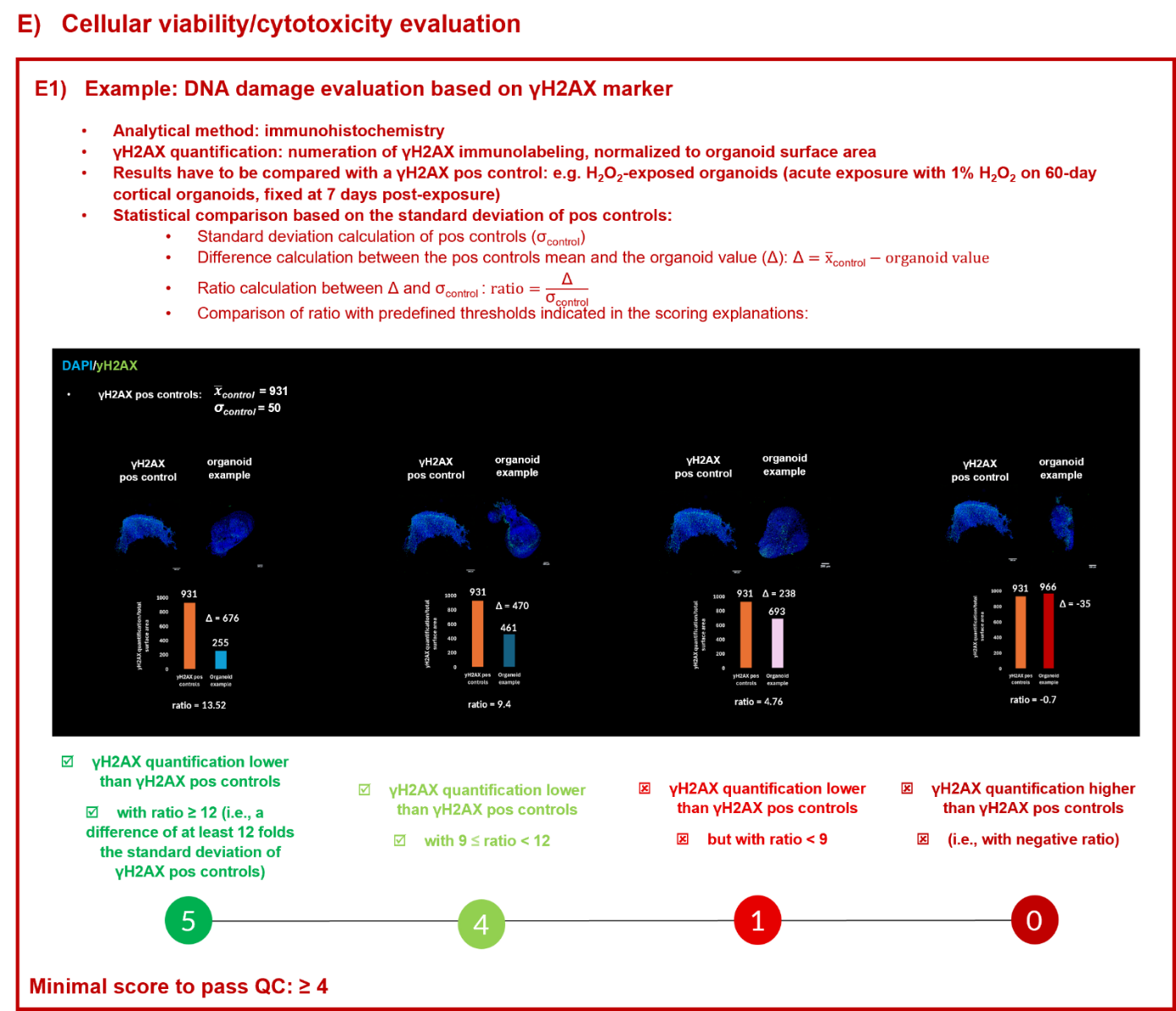
**

**Fig. S1.** Quality Control (QC) of 60-day cortical organoids, based on a scoring methodology**.**

Detailed version of the QC scoring system, including the five primary criteria and sub-indices for organoid evaluation, accompanied by illustrative examples associated with score thresholds. Analytical methods and notes for certain criteria enable to facilitate the utilization and transposition of the scoring. The minimal required QC score for each index is also mentioned.


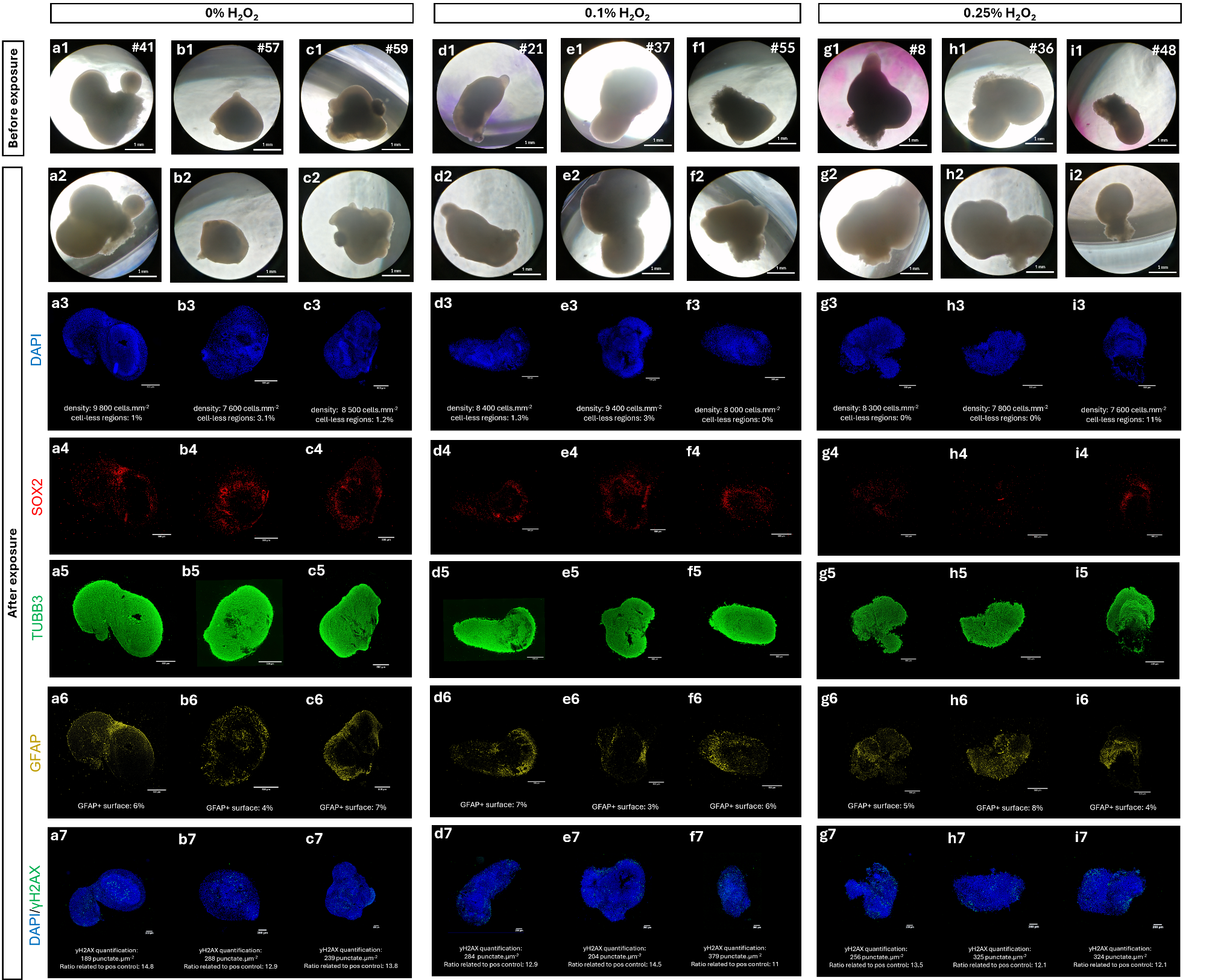


**Fig. S2.** Illustrative images of all cortical organoids after H_2_O_2_ exposures (first part of the figure, to be continued in Fig. S3).

Quality Control (QC) for evaluation of cortical organoids after H_2_O_2_ exposures, following the Final Scoring based on all the criteria. (**a1-i1**) Organoids exposed to different H_2_O_2_ doses comprised between 0% and 0.25%. Morphology before (**a1-i1**) and after (**a2-i2**) H_2_O_2_ exposures serve to evaluate the first criterion related to morphological quality evaluation (brightfield, 5X). Immunofluorescent staining for DAPI (**a3-i3**), neural progenitor marker SOX2 (**a4-i4**), neuronal marker TUBB3 (**a5-i5**), and astrocytic marker GFAP (**a6-i6**) enable the assessment of the following criteria: verification of cell types presence, assessment of astrocytic reactivity, and evaluation of cytoarchitectural organization. Immunofluorescent labeling of DNA damage with yH2AX marker enables evaluation of cytotoxicity level (**a7-i7**) (Leica THUNDER microscope, 20X). Additional data on the other H_2_O_2_ exposure doses can be found in Fig. S3.


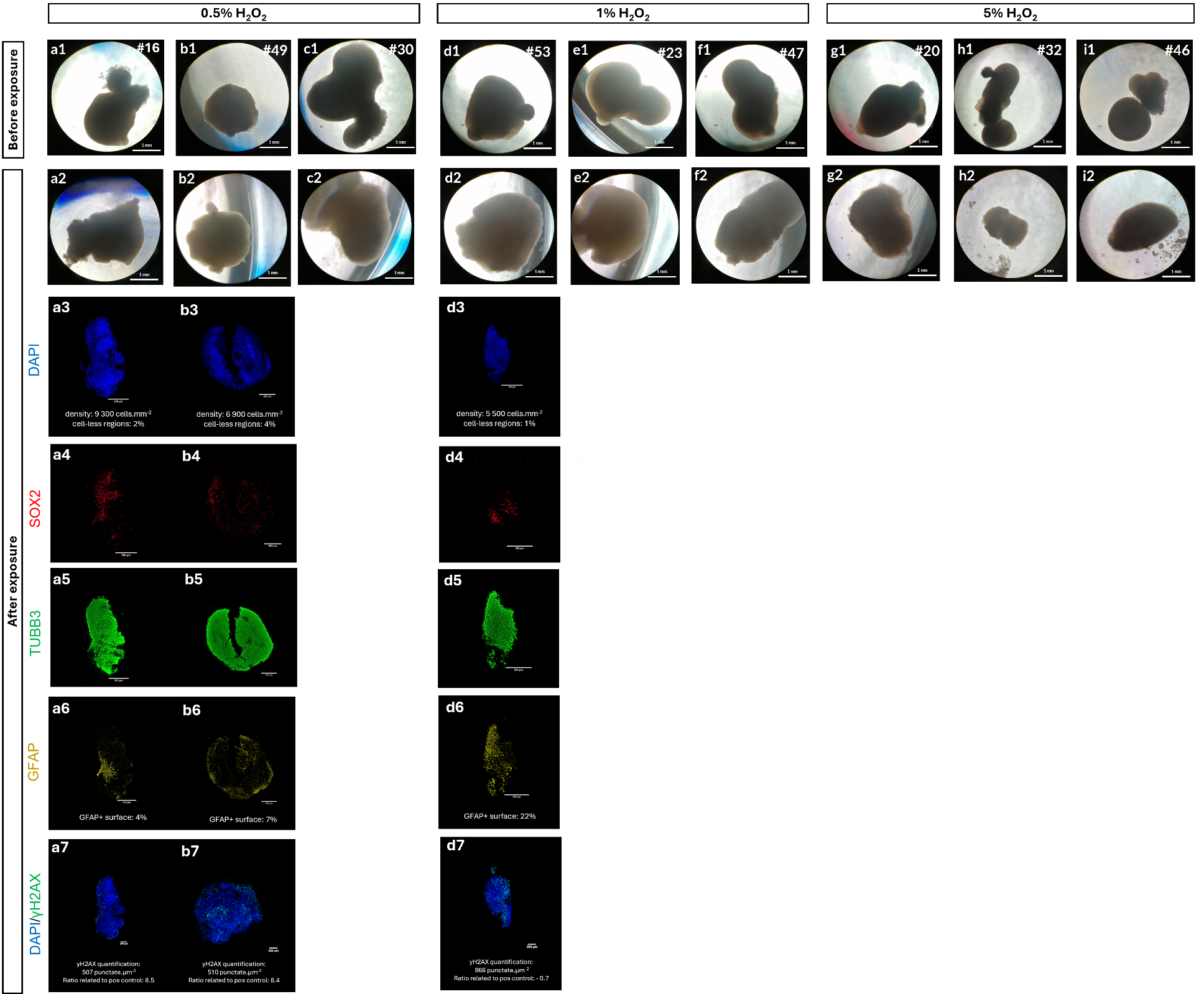


**Fig. S3.**Illustrative images of all cortical organoids after H_2_O_2_ exposures (second part of the figure).

Quality Control (QC) for evaluation of cortical organoids after H_2_O_2_ exposures, following the Final Scoring based on all the criteria. (**a1-i1**) Organoids exposed to different H_2_O_2_ doses comprised between 0.5% and 5%. Morphology before (**a1-i1**) and after (**a2-i2**) H_2_O_2_ exposures serve to evaluate the first criterion related to morphological quality evaluation (brightfield, 5X). Immunofluorescent staining for DAPI (**a3-d3**), neural progenitor marker SOX2 (**a4-d4**), neuronal marker TUBB3 (**a5-d5**), and astrocytic marker GFAP (**a6-d6**) enable the assessment of the following criteria: verification of cell types presence, assessment of astrocytic reactivity, and evaluation of cytoarchitectural organization. Immunofluorescent labeling of DNA damage with yH2AX marker enables evaluation of cytotoxicity level (**a7-d7**) (Leica THUNDER microscope, 20X). Organoids for which the immunofluorescent staining images are not presented correspond to those that could not undergo embedding and cryosectioning processing due to insufficient density and compactness. Additional data on the other H_2_O_2_ exposure doses can be found in Fig. S2.


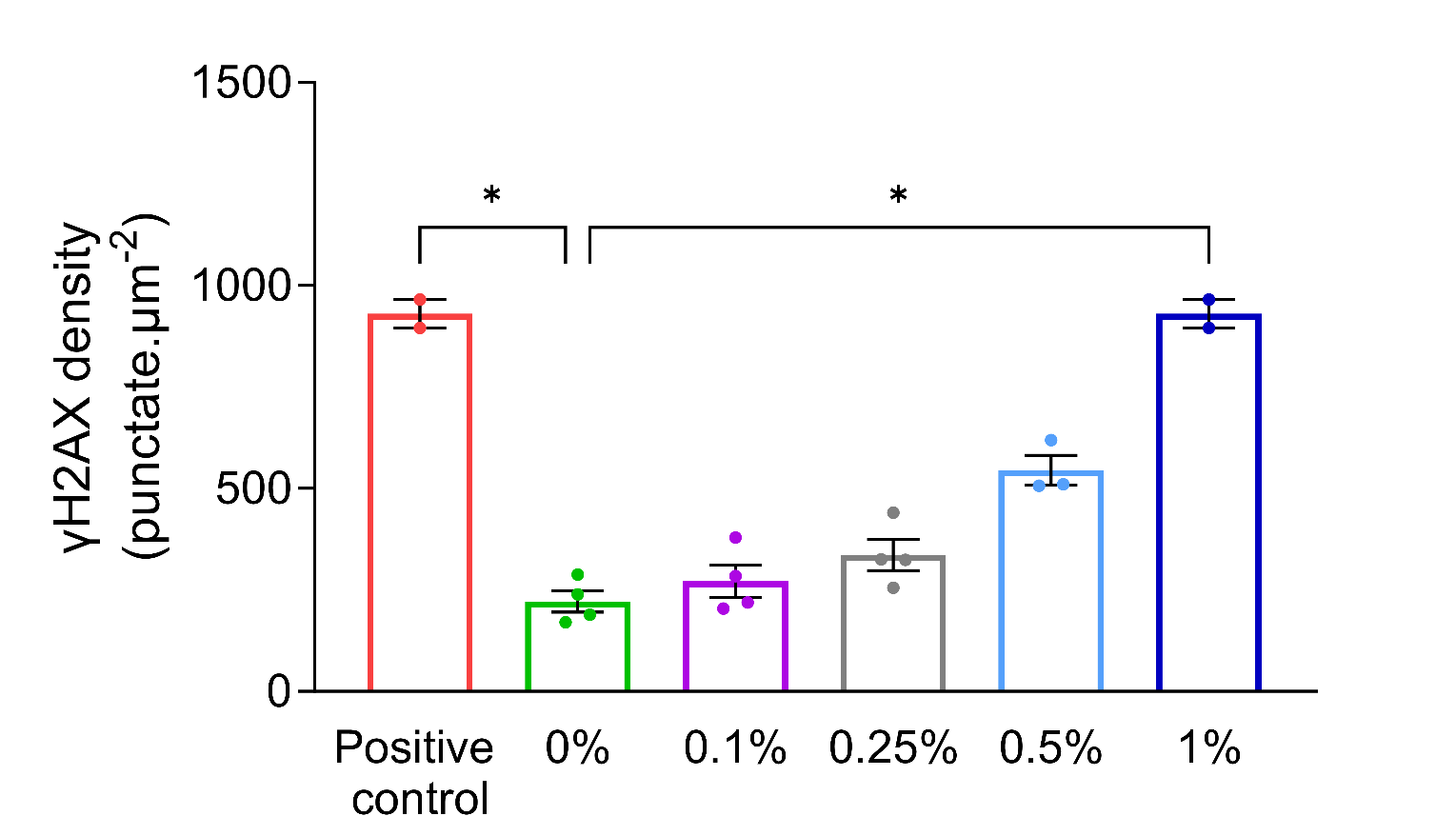


**Fig. S4.** Cytotoxicity evaluation index for H_2_O_2_-exposed organoids.

Bar plot representing the γH2AX quantification in the organoids exposed to varying H_2_O_2_ concentrations between 0% and 1%. γH2AX quantification is expressed in punctate.µm^2^. Positive control organoids and 1%-treated organoids display increased DNA damage compared to control organoids. Kruskal-Wallis test, followed by Dunn’s post-hoc; n=4; p_positive control_ *_vs._* _0%_ = 0.0278; p_0%_ *_vs._* _1%_ = 0.0278.

**Table S1.** Recapitulative table of QC scores obtained for H_2_O_2_-exposed cortical organoids.

| **H_2_O_2_ dose** | **Organoid number** | **A) Morphology** | | | **B) Size and Growth** | | **D) Cellular populations** | | **D) Cytoarchitectural organization** | | | | | **E) Cellular viability / Cytotoxicity** | **Total score** | **QC Final Scoring result** |
| --- | --- | --- | --- | --- | --- | --- | --- | --- | --- | --- | --- | --- | --- | --- | --- | --- |
|  |  | **A1) Density** | **A2) Border integrity** | **A3) Cysts** | **B1) Size** | **B2) Growth** | **C1) Expected cell types** | **C2) Astrocytic reactivity** | **D1) Cellular density** | **D2) Cell-less regions** | **D3) Border integrity** | **D4) Internal cysts** | **D5) Rosettes** | **E1) DNA damage** |  |  |
|  |  | **Minimal score** | | | | | | | | | | | | | |  |
|  |  | 3 / 5 | 2 / 5 | 3 / 5 | 3 / 5 | 3 / 5 | 4 / 5 | 4 / 5 | 3 / 5 | 3 / 5 | 3 / 5 | 3 / 5 | 3 / 5 | 4 / 5 | 35 / 50 |  |
| **0%** | **#11** | 5 | 5 | 5 | NA | | 5 | 5 | 5 | 5 | 5 | 5 | NA | 5 | 50 | **Passed** |
|  | **#41** | 5 | 4 | 5 |  |  | 5 | 5 | 4 | 4 | 5 | 5 |  | 5 | 47 | **Passed** |
|  | **#57** | 5 | 5 | 5 |  |  | 5 | 5 | 3 | 4 | 5 | 5 |  | 5 | 47 | **Passed** |
|  | **#59** | 5 | 5 | 5 |  |  | 5 | 5 | 3 | 4 | 5 | 5 |  | 5 | 47 | **Passed** |
| **0.1%** | **#21** | 5 | 5 | 5 |  |  | 5 | 5 | 3 | 4 | 3 | 5 |  | 5 | 45 | **Passed** |
|  | **#37** | 5 | 5 | 5 |  |  | 5 | 5 | 4 | 4 | 5 | 5 |  | 5 | 48 | **Passed** |
|  | **#55** | 5 | 4 | 5 |  |  | 5 | 5 | 3 | 5 | 3 | 5 |  | 5 | 45 | **Passed** |
|  | **#58** | 5 | 5 | 4 |  |  | 5 | 5 | 3 | 5 | 5 | 5 |  | 5 | 47 | **Passed** |
| **0.25%** | **#8** | 5 | 4 | 5 |  |  | 5 | 5 | 3 | 5 | 3 | 5 |  | 5 | 45 | **Passed** |
|  | **#36** | 5 | 4 | 5 |  |  | 5 | 5 | 3 | 5 | 3 | 5 |  | 5 | 45 | **Passed** |
|  | **#48** | 5 | 4 | 5 |  |  | 5 | 5 | 3 | 3 | 3 | 5 |  | 5 | 43 | **Passed** |
|  | **#50** | 5 | 5 | 5 |  |  | 5 | 5 | 3 | 5 | 3 | 5 |  | 4 | 45 | **Passed** |
| **0.5%** | **#16** | 5 | 3 | 5 |  |  | 5 | 5 | 4 | 4 | 3 | 5 |  | *1 🡪 QC failed* | 40 | Failed |
|  | **#30** | 5 | 2 | 5 |  |  | *NA 🡪 QC failed* | x | x | x | x | x |  | x | 12 | Failed |
|  | **#49** | 5 | 5 | 5 |  |  | 5 | 5 | *0 🡪 QC failed* | x | x | x |  | x | 15 | Failed |
|  | **#52** | 5 | 4 | 5 |  |  | 5 | 5 | *0 🡪 QC failed* | x | x | x |  | x | 24 | Failed |
| **1%** | **#23** | 5 | 4 | 5 |  |  | *NA 🡪 QC failed* | x | x | x | x | x |  | x | 14 | Failed |
|  | **#47** | 5 | 4 | 5 |  |  | *NA 🡪 QC failed* | x | x | x | x | x |  | x | 14 | Failed |
|  | **#51** | 5 | 3 | 5 |  |  | 5 | *0 🡪 QC failed* | x | x | x | x |  | x | 18 | Failed |
|  | **#53** | 5 | 4 | 5 |  |  | 5 | *1 🡪 QC failed* | x | x | x | x |  | x | 20 | Failed |
| **5%** | **#17** | 3 | *0 🡪 QC failed* | x |  |  | x | x | x | x | x | x |  | x | 3 | Failed |
|  | **#20** | 5 | *1 🡪 QC failed* | x |  |  | x | x | x | x | x | x |  | x | 6 | Failed |
|  | **#32** | 5 | *0 🡪 QC failed* | x |  |  | x | x | x | x | x | x |  | X | 5 | Failed |
|  | **#46** | 5 | *0 🡪 QC failed* | x |  |  | x | x | x | x | x | x |  | X | 5 | Failed |

Summary table of scores obtained by all the H_2_O_2_-exposed cortical organoids for each criterion and index of the QC. Minimal scores per index and total minimal score required for QC validation are mentioned. Individual final QC scores obtained for each organoid, as well as median scores obtained per H_2_O_2_ dose conditions, are indicated. In addition, the final QC result is mentioned as QC passed/failed.
